# Effective adsorptive removal of triclosan from water using bio-nanocomposite hydrogel beads

**DOI:** 10.3389/fchem.2025.1547169

**Published:** 2025-04-11

**Authors:** Vuyo Moses Mollo, Mthokozisi Mnguni, Diseko Boikanyo, Philiswa Nosizo Nomngongo, James Ramontja

**Affiliations:** ^1^ Department of Chemical Sciences, University of Johannesburg, Johannesburg, South Africa; ^2^ Department of Science and Innovation-National Research Foundation South African Research Chair Initiative (DSI-NRF SARChI) in Nanotechnology for Water, University of Johannesburg, Johannesburg, South Africa

**Keywords:** Triclosan, sodium alginate, manganese sulphide, bio-nanocomposite hydrogels, adsorption removal efficiency, central composite design

## Abstract

**Introduction:**

Triclosan is a common antibacterial drug identified as a major contaminant in South African waters, notably in Gauteng and KwaZulu Natal provinces. This contaminant comes from personal care products and pharmaceuticals. It has been frequently detected in local streams and wastewater treatment plants, posing a threat to aquatic ecosystems and human health. Studies have emphasised the necessity of addressing the presence of triclosan in water bodies to lessen its harmful impacts on the environment.

**Methods:**

In this study, NaAlg/MnS*x* bio-nanocomposite hydrogel beads incorporated with different amounts of MnS NPs (0.02–0.2 g) were synthesised via the ionic gelation method and employed as an adsorbent for the removal of triclosan from aqueous solutions. The surface charge, morphology, thermal stability, crystallinity, and functional groups of NaAlg/MnS bio-nanocomposite hydrogel beads were characterised by SEM equipped with EDX, TEM, Thermogravimetric analysis, FTIR, XRD, and zeta sizer (mV).

**Results and discussions:**

The experimental results demonstrated that incorporating 0.02–0.2 g of MnS NPs in the bio-nanocomposite hydrogels led to enhanced mechanical structure, porosity, and swelling ability for the adsorption of triclosan compared to pristine NaAlg hydrogel. The response surface methodology was used to optimise the experimental parameters affecting the batch adsorption of triclosan onto the surface of the adsorbent. Basic pH conditions were suitable for removing triclosan in aqueous solutions via hydrogen bonding with the carboxyl functional groups of the bio-nanocomposite beads. The pseudo-second order, Freundlich, and Sips models better explained the adsorption kinetics and equilibrium isotherm data. The maximum adsorption capacity estimated using the Langmuir isotherm model was 132 mg/g. The thermodynamic parameters (enthalpy (∆H) and entropy (∆S)) were found to be 44.042 kJ/mol and 207.018 J/Kmol, respectively, which means the reaction is endothermic and increases randomisation at the solid/liquid interface. The Gibbs free energy (∆G) was negative throughout the studied temperature range, indicating that the adsorption process was spontaneously and energetically favoured.

## 1 Introduction

Triclosan (C_12_H_7_Cl_3_O_2_, 5-chloro-2-(2,4-diclorophenoxy) phenol) is a widely applied preservative and antibacterial compound around the globe. It is mainly used in personal care products, pharmaceuticals, and domestic products due to its excellent response and skin-friendliness. Triclosan is a common ingredient in daily personal care and domestic products such as soap, detergents, disinfectants, toothpaste, mouthwash, plastic additives, shampoos, and deodorants (Luo et al., 2019). After use, triclosan ends up in wastewater treatment plants (WWTPs) through sewage and is usually released with effluents to surface water since they were not designed to treat these emerging contaminants. When introduced into freshwater systems, it creates substantial environmental and health problems by causing harm to aquatic ecosystems, animals, and humans, with algae being the most affected by its toxicity in aquatic systems (Yueh and Tukey, 2016; Olaniyan et al., 2016). Triclosan can also cause cytotoxicity, dermatitis, and bioaccumulation in animal tissues (Qian et al., 2021; Olaniyan et al., 2016). Studies have indicated that excessive exposure to triclosan in fish disrupts their endocrine system, adversely affecting growth, reproduction, and development (Dar et al., 2022; Lee et al., 2024). Triclosan has been detected in various parts of the world, including South Africa. It is found in Gauteng and KwaZulu Natal provinces at concentrations of 1.91–73.5 ug/L in influent samples and 1.732–6.98 ug/L from effluent samples (Musee, 2018; Bakare and Adeyinka, 2022). Therefore, there is a need to remove and limit the amount of triclosan found in freshwater systems and the environment.

Adsorptive removal is emerging as the most used water remediation process because it is easy to operate, flexible, associated with low cost and waste generation, and removal performance.

The adsorptive removal performance and entire adsorption process costs mainly depend on the adsorbent material. Lately, low-cost sorbents for water treatments have been the centre of attention because they offer an extensive alternative to expensive methodologies such as membrane filtration, ion exchange, and carbon adsorption (Reza et al., 2023; Qasem et al., 2021). Adsorbent materials such as activated charcoal, graphene, carbon nanotubes, silica-based materials, and hydrogels have found their way into the water research space (Pereira et al., 2021). Hydrogels are synthesised from bio-resources such as sodium alginate, starch, cellulosic materials, and chitosan, which have grown favourable for environmental use. They have remarkable characteristics that make hydrogels the ideal adsorbent material for removing contaminants in water, such as three-dimensional (3D) porous inner structures, rapid swelling behaviour, and adsorption of large volumes of water (Rizzi et al., 2018; Hossain et al., 2024; Darban et al., 2022). The primary downside of using hydrogels to remove pollutants from water is their poor mechanical strength in their swollen state, which restricts their efficiency and low selectivity (Darban et al., 2022). However, when nanoparticles are incorporated with hydrogels, they can eliminate or minimise most of the disadvantages.

Nanoparticles have several beneficial properties, such as electrical conductivity, mechanical reinforcement, and magnetic properties. Nanocomposite hydrogels offer far more enhanced advantages in removing pollutants from freshwater systems due to their unique features when combined and exhibit synergistic qualities that neither can attain independently (Jiang et al., 2023; Chelu et al., 2023).

Hydrogels can be synthesised using sodium alginate, which is a versatile biomaterial. Alginate is originally found in the cell walls of brown algae, especially from *Laminaria hyperborean, Laminaria digitata, Laminaria japonica, Ascophyllum nodosum, and Macrocystis pyrifera* (Lee and Mooney, 2012). When sodium alginate is cross-linked with divalent cations such as calcium, it forms hydrogels that are incredibly absorbent and can hold a considerable amount of water (Puscaselu et al., 2020; Aderibigbe and Buyana, 2018). Furthermore, sodium alginate hydrogels have a porous structure that provides a wide surface area for adsorption, while their biocompatibility and ease of separation make them ecologically acceptable and cost-effective water treatment materials (Wang and Lu, 2023; Rafiee, 2023; Bustos-Terrones, 2024). On the other hand, MnS NPs offer high adsorption capacity and biocompatibility. They have low toxicity to the environment, with long-term water treatment applications benefiting from the chemical stability that MnS NPs have to offer, and they are easily manufactured at a cheap cost, which is critical for large-scale water treatment applications (Baby et al., 2022; Ajith and Rajamani, 2021). MnS NPs have a large surface area with impressive adsorption properties due to the M-O units on their surfaces (Yang et al., 2019). MnS NPs demonstrate their potential in water treatment procedures, providing a possible alternative to basic adsorbents for tackling contamination in wastewater (Ferretti et al., 2016; Agoro and Meyer, 2022).

Therefore, in this study, the advantages of NaAlg and MnS nanoparticles were combined via an ionic gelation method to successfully synthesise NaAlg/MnS bio-nanocomposite hydrogel beads (at different ratios of MnS nanoparticles) as hybrid adsorbents. Various analytical characterisation techniques systematically characterised the structural and morphological properties and thermal stability. After that, the prepared materials were investigated for their ability to remove triclosan from aqueous solutions. A suitable adsorbent was chosen after investigating the performance (adsorption capacity) of the hybrid bio-nanocomposites and pristine hydrogel. The effect of experimental factors such as (sample volume, mass of adsorbent, and sample pH) affecting the adsorption process was optimised using the response surface methodology (RSM) employing central composite design (CCD).

Furthermore, kinetic, isotherm and thermodynamic experiments of triclosan adsorption onto pristine NaAlg hydrogel and NaAlg/MnS*x* bio-nanocomposite hydrogel beads were investigated by batch experimental mode. Although studies on the removal of triclosan from aqueous media with hydrogel-based adsorbents have been reported in literature, the application of these NaAlg/MnS bio-nanocomposite hydrogel beads to remove triclosan is minimal. Therefore, to our knowledge, the incorporation of MnS NPs onto NaAlg hydrogel beads to remove emerging contaminants such as triclosan has not yet been reported.

## 2 Experimental

### 2.1 Materials and reagents

Sodium alginate (NaAlg), calcium chloride (CaCl_2_), triclosan (TCS), Thioacetamide (TAA) (ACS reagent ≥99%), manganese (II) acetate tetrahydrate ((CH_3_COO)_2_Mn_4_·H_2_O ≥ 99%), sodium hydroxide (NaOH), and acetonitrile (ACN) (HPLC, gradient grade, ≥99.9%) were purchased from Sigma Aldrich Co, Ltd. (St.Loius, MO, USA). Ultra-pure water was prepared by (Direct-Q^®^ 3UV-R purifier system, Millipore, Merck).

### 2.2 Preparation of triclosan stock solution

Triclosan stock solution of 1,000 mg/L was prepared by weighing 100 mg of the solid standard. The weighed triclosan mass was then dissolved in 100 mL acetonitrile and stored in a refrigerator at −4 °C. Distinct concentrations of triclosan were prepared further by diluting 1,000 mg/L triclosan stock solution with acetonitrile.

### 2.3 Synthesis of manganese sulphide nanoparticles (MnS NPs)

The method was adopted from (Jothibas et al., 2017) with minor modifications. MnS NPs were prepared by mixing a (1:3) ratio of manganese (II) acetate and thioacetamide (TAA). Precursors were utilised without any additional purifications. Manganese (II) acetate and thioacetamide (TAA) were mashed separately with a pestle and mortar and, after that, mixed at a ratio of 1:3 g ((CH_3_COO)_2_Mn_4_·H_2_O: TAA). The product of the mixture was calcined in a muffle furnace at 300 °C for 4 h.

### 2.4 Synthesis of pristine NaAlg hydrogel and NaAlg/MnS*x* hydrogel beads

The ionic gelation method was used to synthesise pristine NaAlg hydrogel beads and various formations of NaAlg/MnS*x* bio-nanocomposite hydrogel beads (García et al., 2018). One often-used method for synthesising hydrogels is the ionic gelation process. This approach harnesses the electrostatic interactions between oppositely charged molecules to build structured physical materials such as hydrogel beads.

For this study, an ionic external gelation cross-linking method was used. The polyelectrolyte solution was formulated by dissolving the polymer in water or an appropriate solvent. Thereafter, the polymer solution is subsequently introduced into a beaker/bath containing cross-linking ions such as calcium chloride (CaCl_2_). Upon contact with the cross-linking solution, ions penetrate the polymer droplets, resulting in the gelation process. This process occurs swiftly near the surface, resulting in the creation of a gel shell surrounding the droplet, whereas the core may initially exhibit lower cross-linking (Ahirrao et al., 2014; Gadziński et al., 2023).

The procedure is uncomplicated and does not necessitate specialised apparatus beyond fundamental laboratory tools, and the gelation process does not need high temperatures or harmful chemicals because it takes place under moderate conditions.

### 2.5 Synthesis of pristine NaAlg hydrogel beads

Pristine NaAlg hydrogel beads were prepared by dissolving 3 g of sodium alginate (NaAlg) and 2 g of CaCl_2_ in two distinctive beakers containing 100 mL ultrapure water. The two solutions were stirred at 300 rpm separately at a temperature of 25 °C for 1 h on magnetic stirrers to achieve homogeneous mixtures. After that, the NaAlg solution was added, dropped-wise using a 20 mL syringe, into a stirring (at 300 rpm) CaCl_2_ ionic solution, resulting in the formation of hydrogel beads. The formed NaAlg beads were then left steadily stirring at 200 rpm in the CaCl_2_ solution for 10 min at room temperature (25 °C); after that, they were removed from the magnetic stirrer and left to stay overnight in the CaCl_2_ solution for cross-linking to occur completely. The pristine NaAlg hydrogels were then washed with ultra-pure water to remove excess CaCl_2_ and dried in an oven at 30 °C for 24 h (El et al., 2020).

### 2.6 Synthesis of NaAlg/MnS bio-nanocomposite hydrogel beads

Varying amounts of MnS NPs were mixed with NaAlg solution to synthesise various compositions of NaAlg/MnS bio-nanocomposite hydrogel beads, which were prepared following the exact technique as that for pristine NaAlg hydrogel beads. Briefly, a known amount of MnS NPs (0.02, 0.05, 0.1, and 0.2 g) were added into a stirring (300rpm) NaAlg solution (3 g, 100 mL) at 25°C until the mixture was homogenous. Subsequently, the homogenous mixture was injected using a 20 mL syringe into a CaCl_2_ solution (2g, 100 mL) while stirring at 300 rpm. Once added, the NaAlg/MnS homogenous mixture formed hydrogel beads in the CaCl_2_ solution. The formed NaAlg/MnS bio-nanocomposite hydrogel beads were then left steadily stirring at 200 rpm in the CaCl_2_ solution for 10 min at room temperature (25°C); after that, the formed NaAlg/MnS bio-nanocomposite hydrogel beads were left in the CaCl_2_ solution overnight to cross-link entirely. The NaAlg/MnS bio-nanocomposite hydrogel beads were then washed using ultra-pure water to remove excess CaCl_2_ and dried in an oven at 30°C for 24 h. The nanocomposite hydrogel beads with different amounts of MnS NPs (0.02, 0.05, 0.1, and 0.2 g) were termed NaAlg/MnS*x* bio-nanocomposite hydrogel beads, where “*x*” is the mass of MnS NPs incorporated in the bio-nanocomposite hydrogel beads.

### 2.7 Instrumentation

All pH measurements were made using Bante instruments 901 pH/conductivity meter. Adsorption studies were executed in a Branson 5,800 Ultrasonic bath (Danbury, CT, USA). Scanning electron microscopy (SEM, TESCAN VEGA 3XMU, LMH instrument (Czech Republic)) coupled with energy dispersive x-ray spectroscopy (EDX) using an accelerating voltage of 20 kV was employed to determine the morphology and elemental composition of the adsorbents. Transmission electron microscopic images to measure dimensions were captured using (TEM, JEM-2100, JEOL, Tokyo, Japan). Zeta sizer (ZS, ELS with M3-PALS and Constant Current Zeta Mode, Malvern Panalytical, Westborough, United States). Fourier Transform infrared spectroscopy (FTIR) (Perkin-Elmer Spectrum 100 spectrometer, Perkin-Elmer, USA) was used to study the functional groups of pristine NaAlg and bio-nanocomposite hydrogel beads. Samples were mixed with potassium bromide (KBr) and pressed to form pellets and read at a wavelength of 400–4,000 cm^-1^.

High-performance liquid chromatography (HPLC) 1,200 Infinity series, equipped with a photodiode array detector (Agilent Technologies, Waldbronn, Germany), was used for the analysis of triclosan removal. Separation was achieved using Agilent Zorbax Eclipse Plus C18 column (3.5 µm Χ 150 mm Χ 4.6 mm) Agilent, Newport CA, USA) conditioned at a temperature of 25°C. An injection volume of 5.00 µL, at a flow rate of 1.00 mL/min, at 270 nm adsorption wavelength using a gradient elution system, as seen in Supplementary Table S1.

### 2.8 Zeta potential studies

Zeta potential measurements (mV) were done to understand the behaviour and influence due to electrostatic charges on pristine NaAlg hydrogel and NaAlg/MnS*x* bio-nanocomposite hydrogels. The zeta potential refers to the degree of repulsion between similarly charged particles in a dispersion. The higher the zeta potential, the greater the particle stability, where attraction surpasses repulsion and dispersion (Wu et al., 2018). Zeta potential studies were conducted by weighing 100 mg of pristine NaAlg hydrogel and NaAlg/MnS*x* bio-nanocomposite hydrogels into 500 mL ultrapure water and stirred for 1 h. A volume of 50 mL of the distinctive mixtures was then poured and placed into separate 100 mL beakers, and the pH was adjusted from 2.0 to 10.0. After that, samples were sonicated for 1 h before analysis.

### 2.9 Swelling studies

The swelling studies were carried out to investigate the mechanical swelling ability of the hydrogels. This was achieved by placing 10 mg of material (pristine NaAlg hydrogel and NaAlg/MnS*x* bio-nanocomposite hydrogels) into 100 mL of ultra-pure water at 25 °C for 300 min (5 h). At various intervals from 0–300 min, pristine NaAlg hydrogel and NaAlg/MnS*x* bio-nanocomposite hydrogels were removed and carefully dried using a filter paper, reweighed, and immediately reintroduced into ultra-pure water. Calculations were done according to Equation 1:

$$Swelling\;Ratio\;\%=\frac{W_{wet}-W_{dry}}{W_{wet}}\;Χ\;100\%$$ (1)

where W_dry_ is the mass before, and W_wet_ is the mass after the hydrogels are immersed in ultra-pure water to determine the mass after.

### 2.10 Design of experiment for ultrasonic-assisted optimisation

The primary independent factors influencing the ultrasonic-assisted technique were optimised using design of experiments (DOE) and are presented in Supplementary Tables S2, 3. Parameters were screened using a multivariate optimisation approach, specifically a central composite design (CCD), to ascertain the optimal conditions. A CCD matrix and experiments were represented in the form of a Pareto chart. The chart shows the standardised effect and the interactions between all parameters, which allows the identification of significant factors efficiently. The parameters in the Pareto chart are listed on the left side, while the standardised effect estimates are displayed on the right side. A higher value on the chart implies a more significant influence on the response variable. The red line on the Pareto chart at p = 0.05 represents the degree of significance of the effect. When a standardised impact parameter surpasses the red line, its influence is deemed statistically significant. A total of eighteen experimental runs were established with centre points. The optimisation process involved estimating the response of the fit model, estimating the coefficients by fitting experimental data to the response functions, and analysing the response of statistically selected combinations.

A CCD technique based on response surface methodology (RSM) was applied to optimise factors such as pH, mass of adsorbent (MA), and sample volume (SV). RSM is a statistical and mathematical tool for planning, optimising and comprehending processes by analysing relationships between multiple variables. RSM is useful when dealing with complicated systems where several independent variables influence the outcome. It helps meet specified objectives, lower unpredictability, maximise or minimise reactions, and pursue numerous goals simultaneously (Khuri and Mukhopadhyay, 2010; Dean et al., 2017).

To achieve maximum adsorption, all parameters were simultaneously optimised using the desirability function. The desirability of 0.0, 0.5, and 1.0 indicates minimal adsorption capacity at equilibrium, average adsorption capacity at equilibrium, and maximum adsorption capacity at equilibrium, respectively. The numbers at the bottom of the chart display desirability values for each independent factor (MA, pH, and SV). A target of 1.0 for desirability was used to determine optimal circumstances for maximum adsorption capacity at equilibrium.

### 2.11 Ultrasonic-assisted adsorption experiments

Batch experiments were carried out using the ultrasonic bath to evaluate the adsorption qualities of NaAlg/MnSx bio-nanocomposite hydrogels in the presence of triclosan solution. Central composite design (CCD) was used to optimise the impact of three significant independent parameters: pH, sample volume (SV), and mass of adsorbent (MA). Following the CCD experimental designs, samples with a volume of 9.8–35.1 mL, pH of 5.32–8.68, and a mass of 3.18–36.8 mg, containing1.0 mg/L triclosan aqueous solutions in 100 mL beakers were sonicated for 30 min (25°C ± 2°C) for optimisation. The supernatant was filtered through a 0.22 µm PVDF membrane syringe filter to separate the NaAlg/MnS*x* bio-nanocomposite hydrogels, then injected into 2 mL HPLC vials and measured using HPLC-DAD for initial and equilibrium concentrations. The percentage removal efficiency (%RE) was used to evaluate the results and was calculated using Equation 2:

$$\%\;Removal\;\frac{C_{o}-C_{e}}{C_{o}}Χ100$$ (2)

where C_o_ (mg/L) is the initial concentration of triclosan and C_e_ (mg/L) is the concentration of triclosan at equilibrium.

#### 2.11.1 Adsorption isotherms

Adsorption experiments were conducted to study the adsorption isotherm, kinetic, and thermodynamic aspects of the adsorption process. For adsorption isotherm studies, synthetic solutions of triclosan absorbate were prepared at various concentrations from 5–60 mg/L, while parameters such as sample volume and pH were set at optimal conditions, 35.1 mL and 8.68, respectively. Adsorption isotherms are essential for understanding adsorption equilibrium behaviour and optimising parameters such as adsorbent mass. They give qualitative information on adsorbent uptake capacities and aid in analysing equilibrium adsorption data (Bagal and Raut-Jadhav, 2021). Adsorption isotherms can provide insights into the adsorption process and the type of adsorption, whether monolayer or multilayer adsorption (Murphy et al., 2023). The adsorption capacity (q_e_, mg/g) was calculated using Equation 3:

$$q_{e}=\left(\frac{C_{o}-C_{e}}{m}\right)Χ\;V$$ (3)

where V (mL) is the volume of triclosan in solution and *m* is the mass of NaAlg/MnS*x* hydrogels (mg).

Non-linear adsorption isotherm models such as Temkin, Langmuir, Freundlich, Sips, and Dubinin-Radushkevich (D-R) were fitted to understand the behaviour of the adsorbent Eqs, as shown in Supplementary Table S4.

#### 2.11.2 Adsorption kinetics

Kinetic studies were conducted to determine the effect of contact time (min). The sample containing 60 mg/L triclosan and prepared under optimum conditions was sonicated for 5–85 min. Adsorption kinetics provide vital information on the adsorbent performance and adsorption process mechanism by evaluating contact time (Huang et al., 2021). Equation 4 was used to determine the adsorption capacity (q_t_, mg/g) at a given time:

$$q_{t}=\left(\frac{C_{0-}C_{t}}{m}\right)Χ\;V$$ (4)

where C_t_ (mg/L) is the concentration of triclosan in the solution at the time (min).

Non-linear adsorption kinetic models such as Elovich, intra-particle diffusion, pseudo-first order, and pseudo-second order employed to evaluate the data and Eqs are presented in Supplementary Table S5.

#### 2.11.3 Thermodynamic studies

Thermodynamic experiments were investigated to determine the effect of temperature on adsorption; sample temperatures were varied from 298–318 K to treat 60 mg/L concentration of triclosan under optimum conditions. Thermodynamics gives valuable information on the spontaneity, feasibility, and nature of the adsorption process. Thermodynamic properties like Gibbs free energy change (∆G), enthalpy change (∆H), and entropy change (∆S) are quantified to determine if an adsorption process is spontaneous or non-spontaneous and exothermic or endothermic (Saha and Chowdhury, 2011; Ebelegi et al., 2020). Equations 5–7 were combined to produce a linear Equation 8, in which lnK_d_ was plotted against 1/T. The van’t Hoff plot was used to determine ∆H and ∆S from the slope (-∆H/R) and y-intercept (∆S/R), respectively.

$$K_{d}=\frac{q_{e}}{C_{e}}\;Χ\;\rho$$ (5)

$$∆G=-\text{RTIn}K_{d}$$ (6)

∆G=∆H − T∆S (7)

$${\text{InK}}_{d}=\frac{∆S}{R}-\frac{∆H}{\text{RT}}$$ (8)

where K_d_, *ρ*, T, and R are the adsorption affinity, density of the solution (1,000 g/L), temperature in Kelvin (K), and gas constant (8.314 J/mol.K), respectively.

### 2.12 Reusability studies

To develop an economically viable adsorbent for the removal of triclosan reusability, studies for NaAlg/MnS_0.05g_ bio-nanocomposite hydrogel were conducted. Each cycle included agitating NaAlg/MnS_0.05g_ hydrogel in an ultrasonic bath at optimal conditions, as the initial concentration of triclosan aqueous solutions was 1.0 mg/L. Following efficient adsorption of the triclosan analyte, it was eluted using NaOH solution (0.1 M, 100 mL) for 2 h, followed by rinsing with ultrapure water till neutralisation for reuse (Shoaib et al., 2023).

## 3 Results and discussion

### 3.1 Characterisation of the materials

#### 3.1.1 FTIR analysis

**FIGURE 1:**
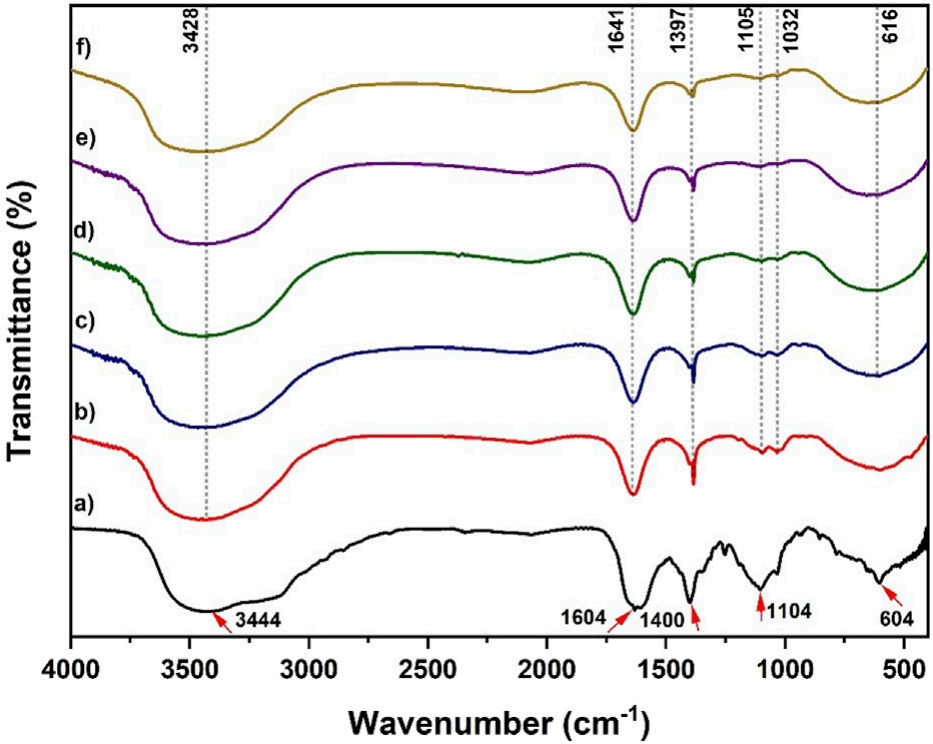
FTIR spectra of **(a)** MnS NPs, **(b)** pristine NaAlg hydrogel, **(c)** NaAlg/MnS_0.02g_ hydrogel, **(d)** NaAlg/MnS_0.05g_ hydrogel, **(e)** NaAlg/MnS_0.1g_ hydrogel, and **(f)** NaAlg/MnS_0.2g_ hydrogel.

Figure 1 shows the FTIR spectra for (a) MnS nanoparticles, (b) pristine NaAlg hydrogel, (c) NaAlg/MnS_0.02g_ hydrogel, (d) NaAlg/MnS_0.05g_ hydrogel, (e) NaAlg/MnS_0.1g_ hydrogel, and (f) NaAlg/MnS_0.2g_ hydrogel. Figure 1a shows peaks (marked using red arrows) at 3,444 cm^-1^ and 1,604 cm^-1^ corresponding to the O-H stretching vibrations (Trivedi et al., 2015). The O-H bonds in MnS NPs result from the manganese hydroxide phase that develops before the complete synthesis of manganese sulphide. The peak at 1,400 cm^-1^ is attributed to the N-H stretching vibration, which is from the synthesis precursor thioacetamide (Mathew et al., 2010). The stretching vibrations at 1,104 cm^-1^ correspond to the S=O functional group on the surface of the MnS nanoparticles, while the peak at 604 cm^-1^ is characteristic of the vibration of an Mn-S bond, confirming the successful formation of MnS nanoparticles (Kandhasamy et al., 2024). Figure 1b shows the FTIR spectrum for pristine NaAlg hydrogel, which exhibits a peak at 3,428 cm^-1^, assigned to the O-H stretching vibration. The asymmetric and symmetric vibrations of carboxyl groups are shown at 1,641 cm^-1^ and 1,397 cm^-1^, respectively (Asadi et al., 2018). On the other hand, the C-O and O-H stretching vibrations were found at 1,105 and 1,032 cm^-1^, respectively (Chaturvedi et al., 2024). The FTIR spectra of NaAlg/MnS_0.02g_ hydrogel, NaAlg/MnS_0.05g_ hydrogel, NaAlg/MnS_0.1g_ hydrogel, and NaAlg/MnS_0.2g_ hydrogel present similar bands which means they have similar functional groups; however, some materials exhibit peaks that have lower intensities from its counterparts depending on the amount of MnS NPs incorporated within the hydrogel. The peaks at 1,397, 1,105, and 1,032 cm^-1^ showed a significant decrease in peak intensity as the amount of MnS NPs incorporated increased, as seen in Figures 1c–f, this is thought to be due to that these functional groups (1,397, 1,105, and 1,032 cm^-1^) are the primary functional groups that facilitate bonding between MnS NPs and pristine NaAlg hydrogel matrix in the formation of nanocomposites. The weakened peak strength is also thought to be due to the dilution effect on the bio-nanocomposite hydrogels. This dilution can cause the infrared absorption peak bands to be less intense and weaker (Ullah et al., 2021). The FTIR spectra of the bio-nanocomposite showed a peak at 616 cm^-1,^ which is assigned to the Mn-S band for Figures 1c–f, respectively (Trivedi et al., 2015).

#### 3.1.2 XRD-analysis

**FIGURE 2:**
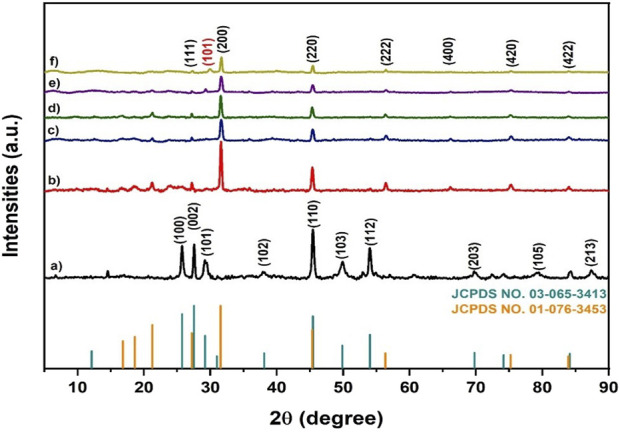
X-ray diffractograms of **(a)** MnS NPs, **(b)** pristine NaAlg hydrogel, **(c)** NaAlg/MnS_0.02g_ hydrogel, **(d)** NaAlg/MnS_0.05g_ hydrogel, **(e)** NaAlg/MnS_0.1g_ hydrogel, **(f)** NaAlg/MnS_0.2g_ hydrogel, and JCPDS card number 03-65-3413 and 01-076-3453.

Figure 2 dissipates X-ray diffractograms for MnS nanoparticles, pristine NaAlg hydrogel, and the NaAlg/MnS*x* bio-nanocomposite hydrogels. Figure 2a shows sharp diffraction peaks at 2θ = 25.80°, 27.55°, 29.28°, 38.13°, 45.40°, 49.91°, 54.07°, 69.84°, 79.22°, and 87.36° that correspond to the faces (100), (002), (101), (102), (110), (103), (112), (203), (105), and (213) of MnS NPs, respectively. The XRD patterns of Figure 2a are similar to those of JCPDS No. 03–065–3,413. A comparison with the JCPDS file verifies the existence of a certain crystalline phase in the sample. The diffraction pattern of each material is distinctive and functions as a “fingerprint” for identification. The phase present in the sample is determined by comparing the relative intensities and positions of the diffraction peaks (2θ values) to the database, in this instance, the JCPDS file.

The diffraction peaks of Figure 2b at 2θ were in 31.59°, 45.36°, 56.31°, 66.16°, 75.27°, and 83.99° that correspond to the faces (111), (200), (220), (222), (400), (420), and (422), respectively, as they were confirmed by JCPDS No. 01–076–3,453. The identification of the diffraction peaks confirmed the successful synthesis of pristine NaAlg hydrogel. The addition of MnS NPs onto pristine NaAlg hydrogel is expected to bring changes to the XRD pattern of pristine NaAlg hydrogel, such as peak broadening, intensity variations, phase composition alterations, adjustments in lattice parameters, and preferred orientation effects. For instance, the introduction of nanoparticles may result in a change in the intensity of diffraction peaks. This may be ascribed to several reasons, such as the preferred orientation by MnS NPs, which can amplify certain peaks or diminish others. Furthermore, fluctuation in the quantity of MnS NPs might influence peak intensities (Holder and Schaak, 2019; Chhantyal, 2022).

Figures 2c–f shows the XRD patterns of NaAlg/MnS*x* bio-nanocomposite hydrogels. They had shown 2θ diffraction peaks to pristine NaAlg hydrogel, but the peak intensities of NaAlg/MnS*x* bio-nanocomposite hydrogels were much lower. The low intense peaks from NaAlg/MnS*x* bio-nanocomposite hydrogels are due to the introduction of MnS NPs on the surface of pristine NaAlg gel, which then loses its crystallinity. Moreover, this phenomenon also indicates a structural shift within the NaAlg/MnS*x* bio-nanocomposite hydrogels, which also suggests a successful synthesis of bio-nanocomposite hydrogels (Júnior et al., 2021).

XRD pattern of NaAlg/MnS_0.02g_ hydrogel and NaAlg/MnS_0.05g_ hydrogel, Figures 2c, d did not reveal any diffractions linked to MnS NPs phase, indicating that either there is a low mass of MnS NPs phase present with the nanocomposite hydrogel or that MnS NPs are entirely wrapped inside the nanocomposite hydrogel. However, as mass of MnS NPs was increased to form NaAlg/MnS_0.1g_ hydrogel and NaAlg/MnS_0.2g_ hydrogel, the diffraction peak at 2θ = 29.28° for the face (101) corresponding to MnS NPs was revealed and confirmed using JCPDS No. 03-065-3413. This indicates that the properties and structural characteristics of MnS NPs are present in the synthesised bio-nanocomposite hydrogels (Agoro and Meyer, 2022).

#### 3.1.3 SEM analysis and EDX analysis

**FIGURE 3:**
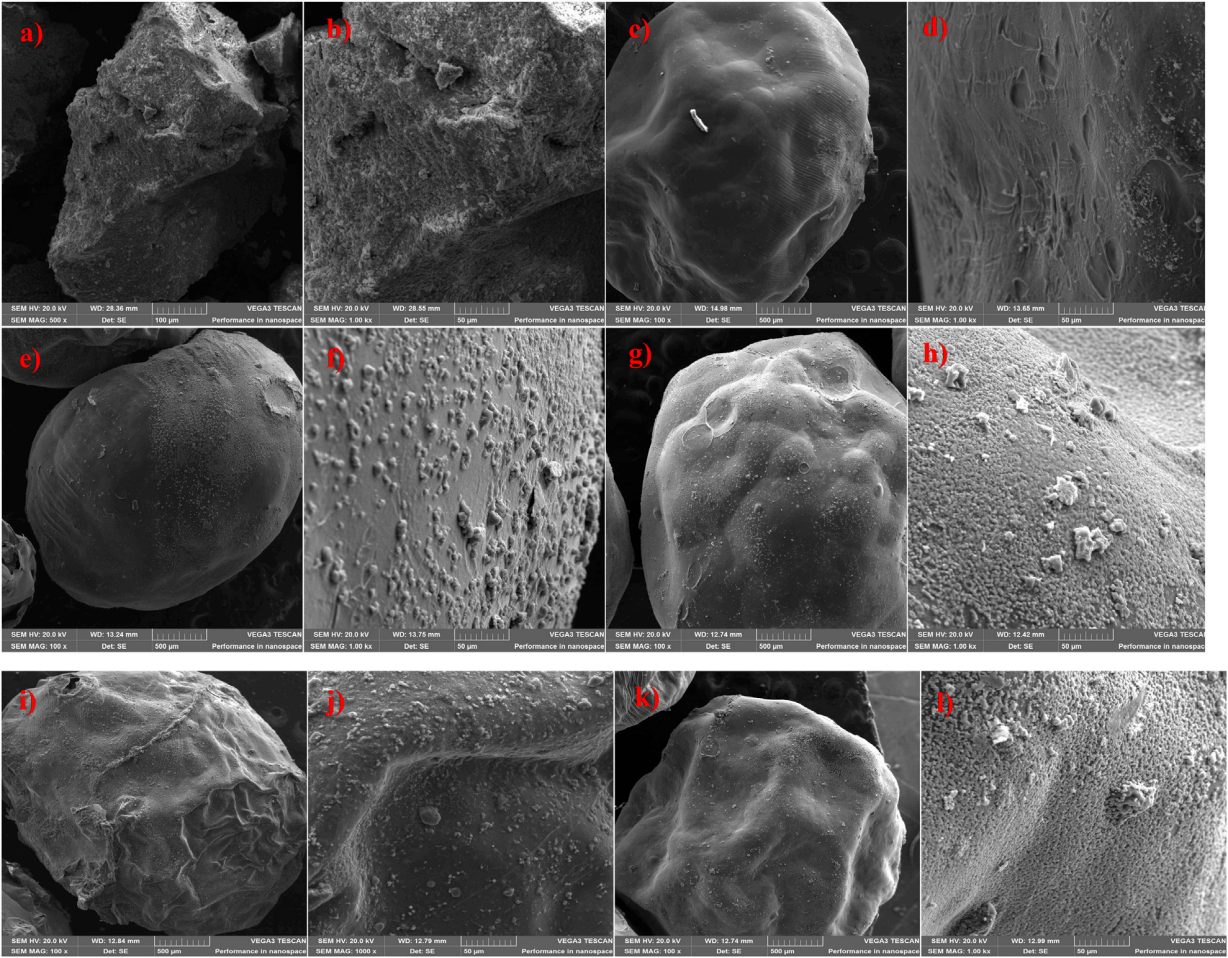
SEM imaging of **(a, b)** MnS NPs; **(c, d)** Pristine NaAlg hydrogel; **(e, f)** NaAlg/MnS0.02 g hydrogel; **(g, h)** NaAlg/MnS0.05 g hydrogel; **(i, j)** NaAlg/MnS0.1 g hydrogel; and **(k, l)** NaAlg/MnS0.2 g hydrogel.

Figures 3a–l illustrates the surface morphology of MnS NPs, pristine NaAlg hydrogel, and NaAlg/MnS*x* bio-nanocomposite hydrogels at various magnifications. The SEM imaging showed that all hydrogels were spherical and round-like in shape. Figures 3c, d shows pristine NaAlg hydrogel and a smooth surface with wrinkles and bumps surrounding it. The surface morphology of NaAlg/MnS*x* bio-nanocomposite hydrogels appears to be consistent across all various bio-nanocomposites when viewed using SEM because of the method and approach used to incorporate the nanoparticles into the hydrogel matrices, as seen in Figures 3e–l. For the reduction of particle agglomeration, nanoparticles were synthesised within the hydrogel matrix to ensure a homogenous mixture (Wang and Yao, 2023). NaAlg/MnS*x* bio-nanocomposite hydrogels had a rough outer layer surface surrounded by small stones, which are MnS NPs wrapped and incorporated within and around each bio-nanocomposite hydrogel. SEM also showed that the surface morphology of each bio-nanocomposite is denser, thicker, and rougher at the edges compared to pristine NaAlg. It is also noticeable that folds were visible in each NaAlg/MnS*x* bio-nanocomposite hydrogel. With an increased amount of MnS NPs added to each bio-nanocomposite hydrogel, it can be seen that a larger surface area of nanoparticles is visible around each bio-nanocomposite hydrogel.

**FIGURE 4:**
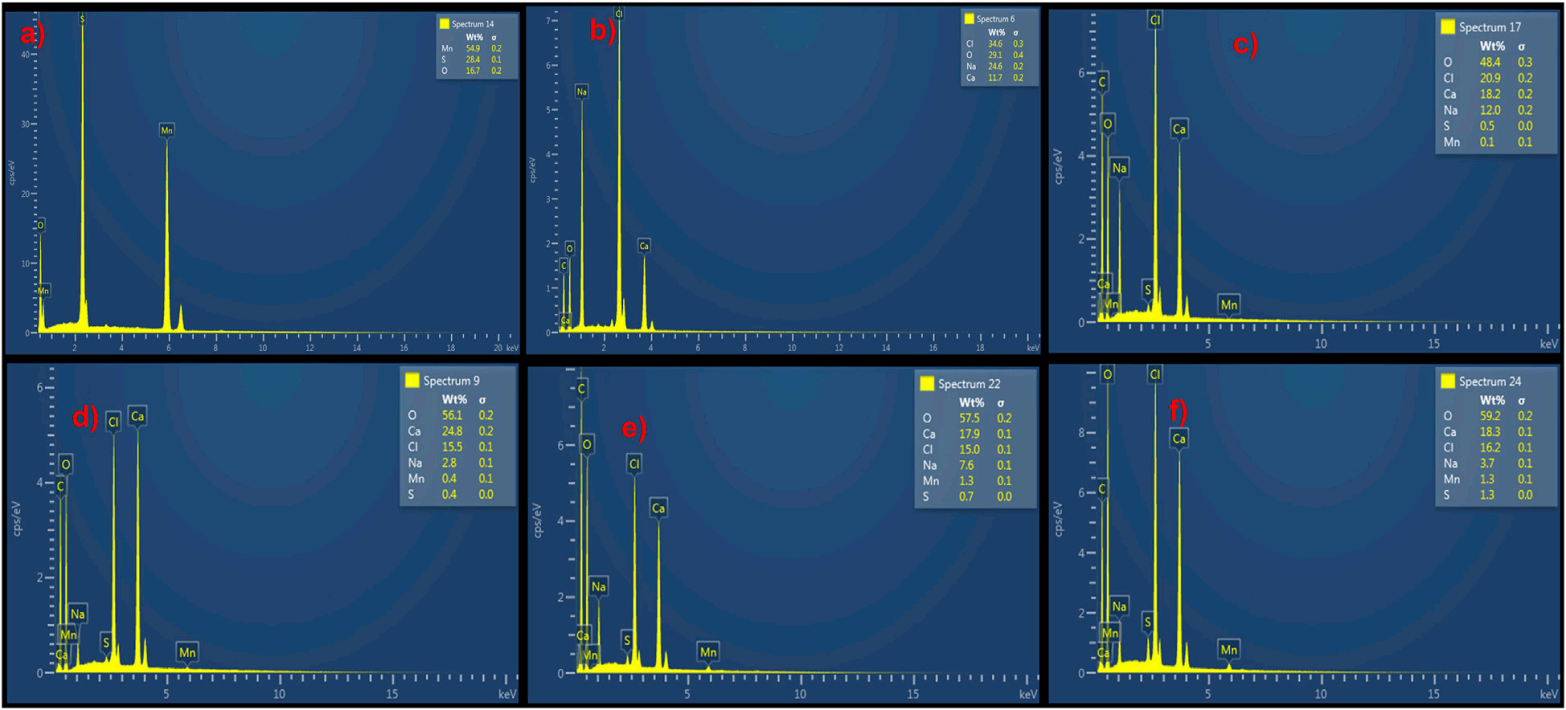
EDX elemental composition of **(a)** MnS NPs, **(b)** pristine NaAlg gel, **(c)** NaAlg/MnS_0.02g_ hydrogel, **(d)** NaAlg/MnS_0.05g_ hydrogel, **(e)** NaAlg/MnS_0.1g_ hydrogel, and **(f)** NaAlg/MnS_0.2g_ hydrogel.

The elemental composition of MnS NPs, pristine NaAlg hydrogel, and NaAlg/MnS*x* bio-nanocomposite hydrogels are shown in Figures 4a–f, which were obtained from the SEM images in Figure 3. A successful synthesis of MnS NPs was confirmed by the presence of Mn, S, and O peaks, which are consistent with the chemical composition of MnS NPs, Figure 4a. The spectrum of pristine NaAlg hydrogel showed peaks attributed to Na, Ca, C, O, and Cl peaks, which confirmed it was successfully synthesised. The elemental composition of NaAlg/MnS*x* bio-nanocomposite hydrogels presented in Figures 4c–f include Na, Ca, C, O, Mn, S, and Cl, originating from MnS NPs and pristine NaAlg hydrogel, thus confirming a successful synthesis of the bio-nanocomposite hydrogels. The Cl detected here emanates from the CaCl_2_ solution used to cross-link during the preparation process (Jiang et al., 2017). Chlorine (Cl) may appear in the EDX spectrum of pristine NaAlg hydrogel and NaAlg/MnS*x* bio-nanocomposite hydrogels, but it does not significantly contribute to the enhancement of triclosan adsorption. Rather, pristine NaAlg hydrogel and NaAlg/MnS*x* bio-nanocomposite hydrogel’s surface area, electrostatic interactions, porosity, and functional groups have a greater impact on triclosan adsorption. The elemental composition detected for MnS NPs, pristine NaAlg hydrogel, and NaAlg/MnS*x* bio-nanocomposite hydrogels are consistent with the functional groups confirmed by FTIR spectra in Figure 1.

#### 3.1.4 Transmission electron microscopy

**FIGURE 5:**
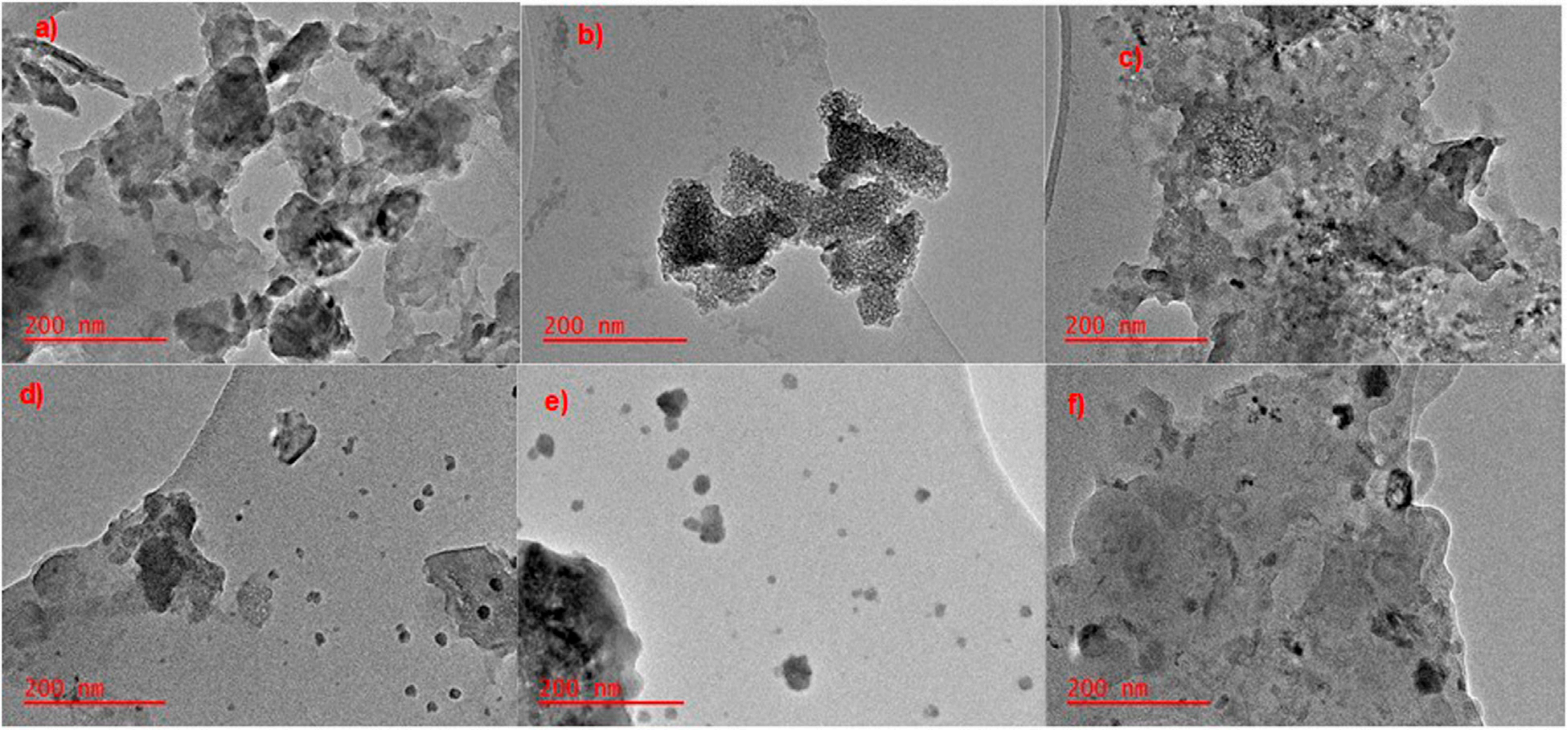
TEM images of **(a)** MnS NPs, **(b)** pristine NaAlg gel, **(c)** NaAlg/MnS0.02g hydrogel, **(d)** NaAlg/MnS0.05g hydrogel, **(e)** NaAlg/MnS0.1g hydrogel, and **(f)** NaAlg/MnS0.2g hydrogel.

Figures 5a–f shows the TEM imaging of MnS NPs, pristine NaAlg hydrogel, and NaAlg/MnS*x* bio-nanocomposite hydrogels. Figure 5a depicts the stacking of MnS NPs, and the darker areas show a bulk stacking of the nanoparticles, while the lighter areas, which are nearly transparent, are thinner layers of MnS NPs. Figure 5b shows the TEM image of pristine NaAlg hydrogel and its layering. The hydrogel appears smooth, with a few dark spots that are thought to be aggregated spots on the hydrogel bulked up on each other. Figures 5c–f shows the TEM image of NaAlg/MnS*x* bio-nanocomposite hydrogels. The distribution and incorporations of MnS NPs nanoparticles within the pristine hydrogel to form NaAlg/MnS*x* bio-nanocomposite hydrogels were consistent and similar when observed. This is due to the ionic gelation synthesis method that formed a homogenous dispersion of nanoparticles throughout each composite and formed similar yet distinct characterisation techniques (Mohamadinia et al., 2021). Figures 5c–f shows each image’s two layers or components of dark and light. The dark, rounded spots are MnS NPs, and the smooth, lighter regions are that of the NaAlg hydrogel matrix. It is noticeable that the bio-nanocomposites appear to have a rougher surface compared to pristine NaAlg hydrogel, which is contributed by the addition of MnS NPs in the hydrogel matrix, creating a heterogeneous surface morphology that is porous (Tsou et al., 2023; Pathania et al., 2020).

#### 3.1.5 Thermogravimetric analysis

**FIGURE 6:**
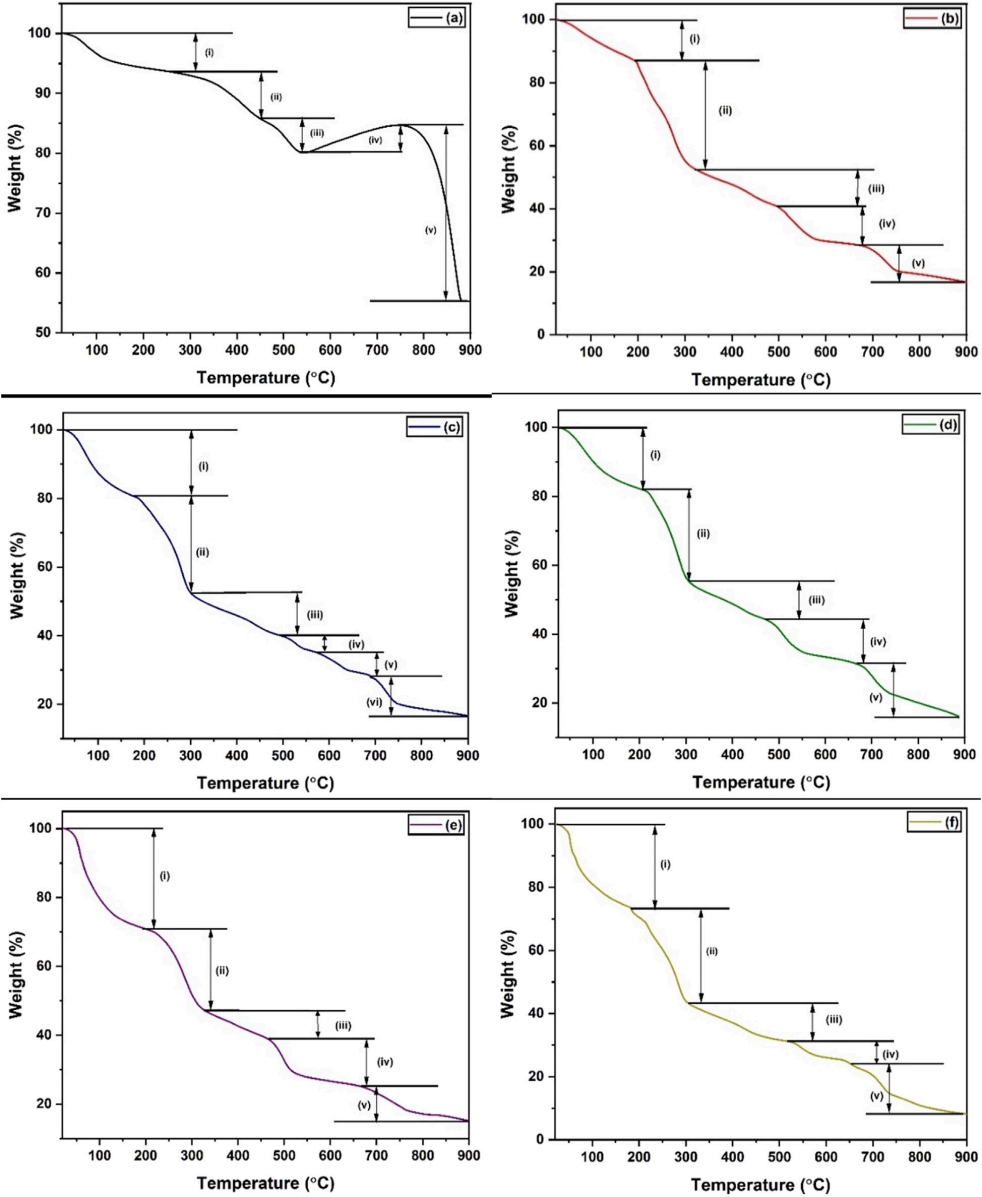
TGA thermographs of **(a)** MnS NPs, **(b)** pristine NaAlg hydrogel, **(c)** NaAlg/MnS_0.02g_ hydrogel, **(d)** NaAlg/MnS_0.05g_ hydrogel, **(e)** NaAlg/MnS_0.1g_ hydrogel, and **(f)** NaAlg/MnS_0.2g_ hydrogel.

Thermogravimetric analysis (TGA) was carried out to investigate the thermal stability of (a) MnS NPs, (b) pristine NaAlg hydrogel, (c) NaAlg/MnS_0.02g_ hydrogel, (d) NaAlg/MnS_0.05g_ hydrogel, (e) NaAlg/MnS_0.1g_ hydrogel, and (f) NaAlg/MnS_0.2g_ hydrogel. The thermograms shown in Figure 6 illustrate several thermal decomposition steps for all materials. Figure 6a, MnS NPs @ 300°C had high residual temperatures ranging from 872°C to 900°C. MnS NPs @ 300°C had a weight loss of 6.428% in the first degradation step (i). In the second degradation step (ii) for MnS NPs @ 300°C, it was noted that there is a change in peak shape ranging from 261.61°C–450.73°C, which is the removal of oxygen-bonded compounds or groups. The third degradation step (iii) for MnS NPs @ 300°C was found at 450.73°C–538.31°C, which is thought to be the elimination of organic matter and amine groups bonded onto the sample. In the fourth degradation step (iv), it was noticed that there was a weight percentage (%) gain for MnS NPs @ 300°C of 4.51% (538.51°C–753.37°C). The significant weight gain is due to the conversion of MnS into MnSO_4_ and Mn_3_O_4_. Previous studies done by (Ma et al., 2021; Camacho et al., 2019) reported similar trends of MnS. During the last degradation stage (v), MnSO_4_ and Mn_3_O_4_ decompose to Mn_2_O_3_ in all MnS NPs @ 300°C (Jeon et al., 2015).

The first degradation step (i), with a weight loss of 12% for pristine NaAlg hydrogel, occurred around 30°C–193°C, which is attributed to the removal of moisture, as seen in Figure 6b. The second degradation step (ii), with a weight loss of 35% 193°C–322°C, may be attributed to the decomposition of heteroatoms (O) and amorphous carbon, which are responsible for the cross-linking and chain immobilisation (da Silva Fernandes et al., 2018). This is consistent with the functional groups that FTIR confirmed in Figure 1, which include O-H and C-O groups. The third degradation step ranged from 322°C–495°C, with a weight loss of 12%, was aligned to the oxidative decomposition of alginate carbon chains and more stable C=O groups, resulting in the formation of sodium carbonate (Na_2_CO_3_) as a byproduct. The fourth degradation step, between 495°C–667°C, is thought to be residues that produce sodium hydrogen carbonate (NaHCO_3_) and carbonised material, which had a weight loss of 13% (Narayanan and Han, 2017). The fifth degradation step is around 667°C–892 °C and is associated with the decomposition of Na_2_CO_3_ and can be seen in the decomposition (Equations 9, 10) below (da Silva Fernandes et al., 2018).

Step 1

$${\text{Na}}_{2}{\text{CO}}_{3}\;\to \;{\text{Na}}_{2}O+{\text{CO}}_{2}$$ (9)

Step 2

$${\text{Na}}_{2}O\;\to \;2\text{Na}+½\;1/2O_{2}$$ (10)

The degradation steps of NaAlg/MnS*x* bio-nanocomposite hydrogels, as seen in Figures 6c–f, took similar yet distinct shapes to that of pristine NaAlg hydrogel at different thermal decomposition temperatures. The first degradation step (i) had a weight loss of 19, 18, 20, and 27% for NaAlg/MnS_0.02g,_ NaAlg/MnS_0.05g_, NaAlg/MnS_0.1g_, and NaAlg/MnS_0.2g_ hydrogels, respectively, which is attributed to the loss of moisture. The second degradation step (ii) was attributed to major backbone breaking down, which had a weight loss of 28, 26, 24, and 30% for NaAlg/MnS_0.02g,_ NaAlg/MnS_0.05g_, NaAlg/MnS_0.1g_, and NaAlg/MnS_0.2g_ hydrogels, respectively. The third degradation step (iii), which is due to the decomposition of alginate, had a weight loss of 12, 11, 8, and 12% for NaAlg/MnS_0.02g,_ NaAlg/MnS_0.05g_, NaAlg/MnS_0.1g_, and NaAlg/MnS_0.2g_ hydrogels, respectively. The fourth (iv) and fifth (v) degradation steps are attributed to the breakdown of complex alginate bonds for NaAlg/MnS_0.02g,_ NaAlg/MnS_0.05g_, NaAlg/MnS_0.1g_, and NaAlg/MnS_0.2g_ hydrogels, respectively. NaAlg/MnS_0.02g_ bio-nanocomposite had an additional sixth degradation step (vi), which is thought to be from the further breaking down of alginate residues and had a weight loss of 12% (Fernández et al., 2021).

#### 3.1.6 Zeta potential

Supplementary Figures S1a–e shows the zeta potential of pristine NaAlg hydrogel and NaAlg/MnS*x* bio-nanocomposite hydrogels measured against pH in the range of 2.0–10.0. The surface charge of pristine NaAlg hydrogel and NaAlg/MnS*x* bio-nanocomposite hydrogels was negative throughout the studied pH range due to the presence of carboxylic groups (Abourehab et al., 2022). The deprotonation of carboxyl groups in the alginate polymer at neutral to basic pH conditions is the primary cause of the negative surface charge of alginate hydrogels (Rosiak et al., 2021; Banks et al., 2019). The incrementing incorporation of MnS NPs had no significant effect on the zeta potential of NaAlg/MnS*x* bio-nanocomposite hydrogels. This is because zeta potential is primarily dependent on the surface charge of the alginate hydrogel that is predominately dictated by metal ions, such as calcium ions, which are responsible for the cross-linking mechanism of the matrix of hydrogel (Hu et al., 2023). Although the surface charge of pristine NaAlg hydrogel and NaAlg/MnS*x* bio-nanocomposite hydrogels is negative, alginate hydrogel matrixes can adsorb triclosan in wastewater. This is because the hydrogel’s capacity to establish hydrogen bonds (Neves et al., 2020; Akshaya and Nathanael, 2024), hydrophobic interactions (Rosiak et al., 2021; Asadi et al., 2018), physical entrapment (Rigoletto et al., 2023; Banks et al., 2019), and electrostatic interactions (Ciarleglio et al., 2023; Abasalizadeh et al., 2020) with divalent cations enables the effective adsorption of triclosan, despite their similar charges. The adsorption capacity is influenced by parameters such as pH, ionic strength, and the presence of organic material, as seen in studies by (Rasoulzadeh et al., 2023; Chaturvedi et al., 2024).

#### 3.1.7 Swelling studies

Supplementary Figures S2a–e shows the swelling properties of pristine NaAlg hydrogel and NaAlg/MnS*x* bio-nanocomposite hydrogels. This study was done to understand the swelling properties and mechanical changes contributed by the addition of MnS NPs onto the NaAlg hydrogel matrix. The swelling study was conducted for 300 min (5 h) and it was noticeable that pristine NaAlg gel had a swelling ratio of 10% at 5 min. The swelling ratio (%) of pristine NaAlg gel was directly proportional to time, and as the time spent by pristine NaAlg hydrogel in ultrapure water, the swelling ratio increased (%). The swelling in pristine NaAlg hydrogel is influenced by the hydrogen bonding of carboxyl (-COOH) and hydroxyl (-OH) groups present in the hydrogel, as confirmed by FTIR in Figure 1 (Liu et al., 2016) due to the presence and abundance of carboxyl (-COOH) and hydroxyl (-OH) groups in NaAlg. NaAlg-based hydrogels are exceedingly hydrophilic, and these functional groups form hydrogen bonds with water molecules, making them responsible for the swelling behaviour of hydrogels (Anugrah et al., 2022).

NaAlg/MnS*x* bio-nanocomposite hydrogels showed excellent swelling properties compared to pristine NaAlg. The superior swelling properties of NaAlg/MnS*x* bio-nanocomposite hydrogels result from their improved network structure, which is a result of the incorporation of MnS NPs, increased porosity, optimised cross-linking, and improved mechanical properties (Zhang et al., 2014; Gao et al., 2020). The study also showed that with an increased amount of incorporated MnS NPs, the greater the swelling ratio: NaAlg/MnS_0.2g_ > NaAlg/MnS_0.1g_ > NaAlg/MnS_0.05g_ > NaAlg/MnS_0.02g_ > pristine NaAlg. However, there was a slight exception for NaAlg/MnS_0.05g_ and NaAlg/MnS_0.1g_ hydrogel, as the swelling ratios (%) were close to each other. These enhanced properties were formed from the cohesion between MnS NPs and the NaAlg hydrogel matrix. MnS NPs also have N-H and O-H functional groups, as seen from the FTIR spectra in Figure 1, which can also promote hydrogen bonding with water and triclosan molecules. Nanoparticles improve the swelling capacity of hydrogels in composites by changing the structure and features of a hydrogel. Nanoparticles incorporated with hydrogels can form wider empty pockets within the matrix, allowing more solvent penetration, which means the greater the solvent penetration between the hydrogel’s polymeric chains, the greater the swelling ability. Furthermore, the inclusion of nanoparticles can influence the mechanical strength of the hydrogel, in this case, expanding its ability to retain liquids (van den et al., 2010; Mohammed et al., 2022; Karchoubi et al., 2023). The increased swelling properties also suggest stronger interactions between NaAlg/MnS*x* bio-nanocomposite hydrogels and triclosan. This may be the result of robust interactions, such as hydrogen bonding, electrostatic interactions, and hydrophobic effects, which facilitate the adsorption of triclosan into the hydrogel matrix (Karadağ et al., 2020; El et al., 2018).

#### 3.1.8 Performance of NaAlg based hydrogels for removal of triclosan

The removal performance of pristine NaAlg hydrogel and NaAlg/MnS*x* bio-nanocomposite hydrogels for triclosan were compared among each other to select the best-performing adsorbent, as seen in Supplementary Figures S3a–e. The removal recoveries of each adsorbent were determined using Equation 3. Supplementary FigureS3a showed that pristine NaAlg hydrogel had the lowest and poorest removal rate of triclosan compared to the NaAlg/MnS*x* bio-nanocomposite hydrogels. Pristine hydrogels generally exhibit lower mechanical strength and stability compared to nanocomposite hydrogels, which benefit from the incorporation of nanoparticles. Nanocomposites provide improved tensile strength, compressive strength, and toughness, rendering them more suitable than pristine hydrogels. Supplementary Figure S3c indicated that NaAlg/MnS_0.05g_ bio-nanocomposite performed effectively well for the removal of triclosan, as it had the highest percentage removal recovery compared to pristine NaAlg hydrogel and the remaining bio-nanocomposites hydrogels. This could be due to the non-uniform distribution of MnS NPs inside the hydrogel, which may result in localised regions exhibiting enhanced or diminished adsorption properties. An evenly dispersed nanoparticle network optimises active sites, whereas aggregation may diminish efficacy (Karchoubi et al., 2023; Gutierrez et al., 2022). Therefore, adsorption studies applied for the removal of triclosan in water were further conducted using NaAlg/MnS_0.05g_ bio-nanocomposite hydrogel.

#### 3.1.9 Optimisation of experimental parameters using a central composite design

Central composite design (CCD) was used to investigate the most influential parameters in the removal of triclosan by NaAlg/MnS_0.05g_ bio-nanocomposite hydrogel, which included pH, sample volume (SV), and mass of adsorbent (MA). Supplementary Figure S4 shows a CCD matrix and experiments represented in the form of a Pareto chart. Based on the results shown in Supplementary Figure S4, the SV and pH have a statistically significant effect as they have passed the red line.

Additionally, the parameter with the greatest impact was SV (mL), followed by pH, and MA (mg). The mass of adsorbent (MA) (mg/L) had a minor impact on the experiment, but it was still an important parameter. Therefore, MA (mg), pH, and SV (mL) were further optimised using response surface methodology (RSM) based on the central composite design (CCD).

**FIGURE 7:**
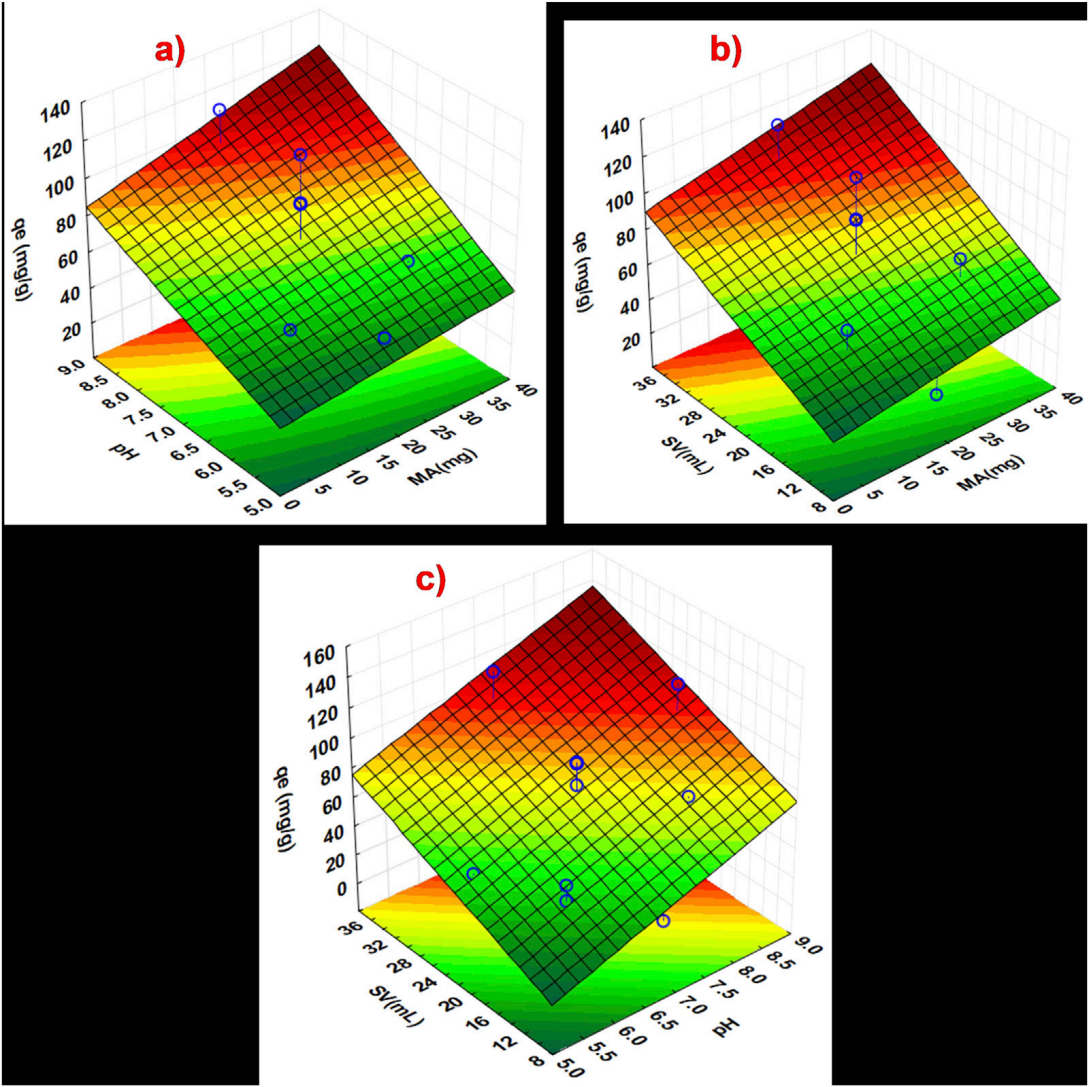
Response surface methodology plots showing the interaction between the independent factors **(a)** MA and pH, **(b)** SV and MA, and **(c)** pH and SV.

A CCD technique based on response surface methodology (RSM) was applied to optimise factors, pH, MA, and SV, as seen in Figures 7a–c. A three-dimensional (3D) surface plot presented in Figures 7a–c shows the cumulative impact of independent variables on the analytical response, including interactions between parameters. The maximum adsorbed concentration at equilibrium (q_e_) for triclosan was achieved when MA was between 11–40 mg in Figures 7a, b. This phenomenon is attributed to the increase in adsorbent dose, which creates a more accessible surface area and adsorption sites for triclosan, thus resulting in better adsorption capacity (Asadi et al., 2018; ALSamman and Sánchez, 2022). The interactive effects for pH showed that between pH 6–9, a better q_e_ (mg/g) was obtained, as seen in Figures 7a, c. The pH affects the surface charge of NaAlg/MnS_0.05g_ bio-nanocomposite hydrogel, as seen in Supplementary Figure S1c. The higher the pH value, the more deprotonated the NaAlg/MnS_0.05g_ bio-nanocomposite hydrogel, which leads to a negative surface charge. However, the adsorption of triclosan by NaAlg/MnS_0.05g_ bio-nanocomposite hydrogel is still possible at pH 8, which is a typical pH range for natural water bodies in the environment (Rigoletto et al., 2023). The impact of SV (mL), when combined with other parameters such as MA and pH, revealed that a SV between 15–35.1 mL is ideal for the experiment. Maintaining an appropriate volume of a sample solution containing triclosan affects the rate of achieving high adsorption capacity (q_e_), leads to improved adsorption efficacy by increasing the contact area and improving mass transfer, and enables superior statistical analysis (Kumari and Gupta, 2019; ALSamman and Sánchez, 2022). The influence of pH, SV, and MA is crucial on the adsorption capacity (q_e_) of NaAlg/MnS_0.05g_ bio-nanocomposite hydrogel in removing triclosan from water.

#### 3.1.10 Desirability function

**FIGURE 8:**
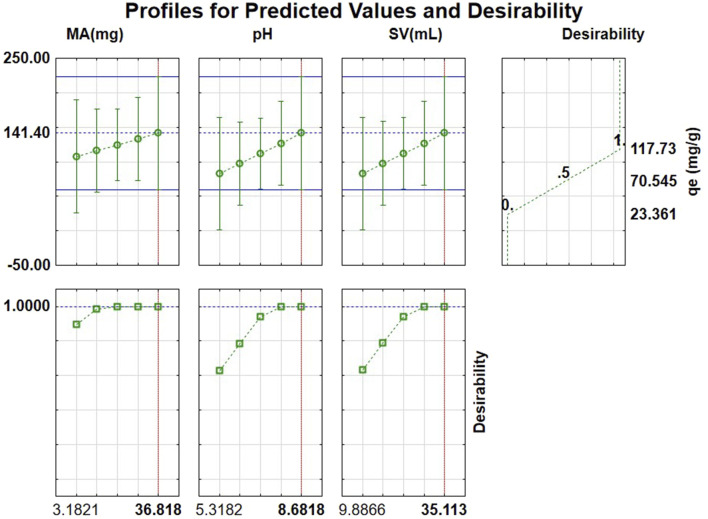
Desirability profiles with predicted values for the investigated factors affecting the adsorption of triclosan.

Figure 8 illustrates the desirability function and anticipated values for determining optimal conditions for the adsorption of triclosan. According to the desirability chart, the optimal pH, SV, and MA adsorption conditions for triclosan were 8.68, 35.1 mL, and 5–36.8 mg, respectively. The chosen mass of adsorbent for the study was 11.5 mg because at a low mass of adsorbent, a higher surface area in relation to the volume and contact with triclosan as the adsorbate results in enhanced adsorption rates. The optimal conditions were tested experimentally at a concentration of 1 mg/L of triclosan, and the tests produced an average adsorption capacity (qe) of 114.1 ± 5.41 mg/g, which is in agreement with the predicted adsorption capacity of 118 mg/g displayed in Figure 8, proving the model valid.

#### 3.1.11 Adsorption studies

##### 3.1.11.1 Adsorption kinetics

**FIGURE 9:**
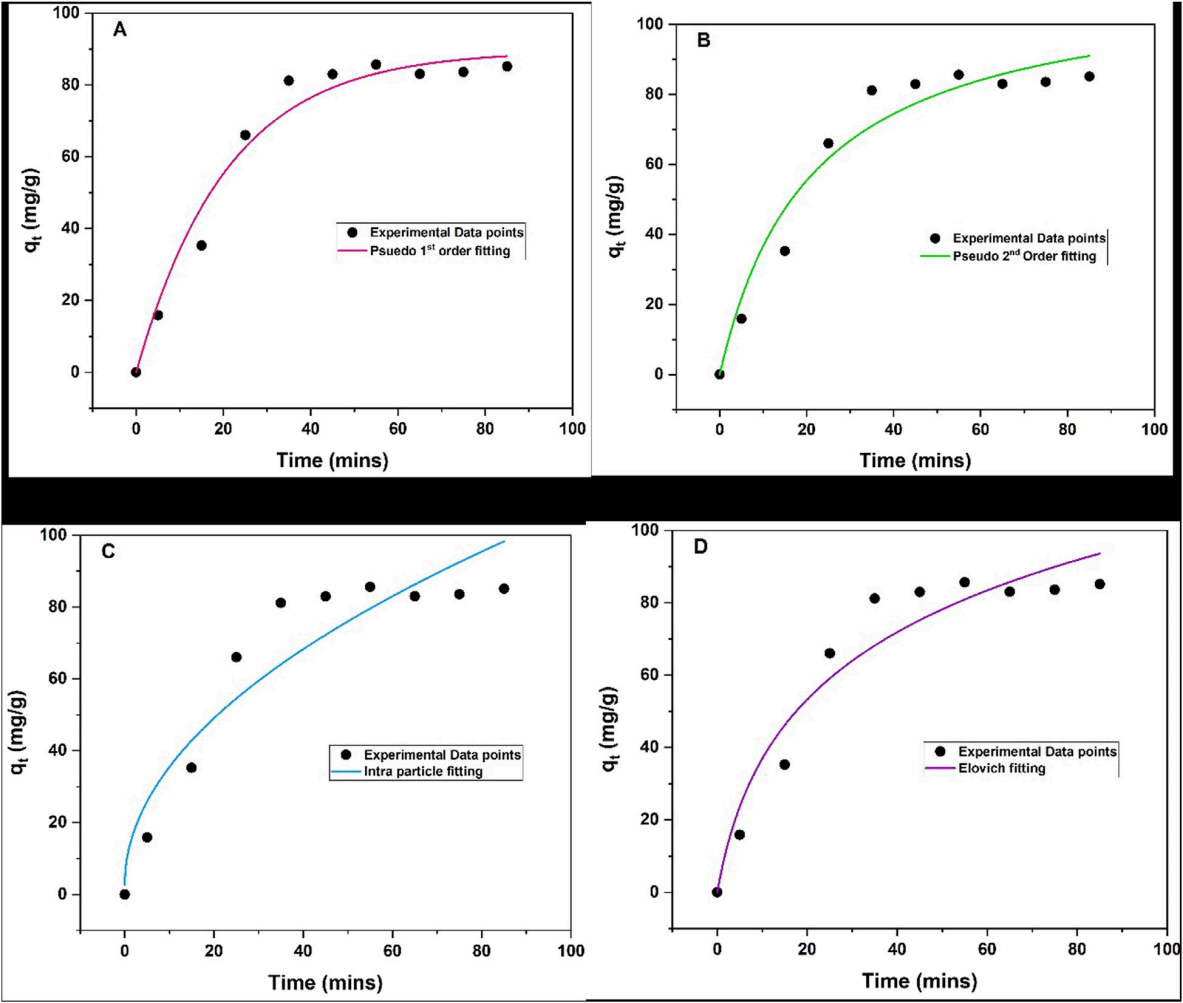
Adsorption kinetic models of NaAlg/MnS_0.05g_ bio-nanocomposite hydrogel on the removal of triclosan **(A)** Pseudo-first order, **(B)** Pseudo-second order, **(C)** Intra-particle diffusion, and **(D)** Elovich model. Experimental conditions: Sample volume, mass of adsorbent, pH, contact time and initial concentration were 35.1 mL, 11.5 mg, 5–85 min 8.68, and 5–60 mg/L.

The adsorption kinetics of triclosan onto NaAlg/MnS_0.05g_ bio-nanocomposite hydrogel were studied using four commonly used kinetic models: pseudo-first order, pseudo-second order, intra-particle diffusion, and Elovich model, and they each have distinct assumptions about an adsorption mechanism. Batch experiments were conducted using 11.5 mg of NaAlg/MnS_0.05g_ bio-nanocomposite hydrogel at an initial concentration of 60 mg/L of triclosan, pH = 8.68, and sample volume of 35.1 mL at room temperature. The adsorbent rapidly absorbed triclosan from 5–35 min (up-take) and reached an equilibrium between 35–85 min, and the graphical data and kinetic models are presented in Figures 9a–d, with the estimated parameters and values given in Table 1. The *R* ^2^ values of all four kinetic models were compared, and the results show that pseudo-second order was favoured because it had the highest *R* ^2^ value. This means that chemical reactions occurring between the triclosan and NaAlg/MnS_0.05g_ bio-nanocomposite hydrogel control the adsorption process and that the adsorption rate is directly proportional to the square of the difference between the equilibrium adsorption capacity and the amount of adsorbate adsorbed at any given time (Tran, 2023; Robati, 2013; Liu et al., 2019).

**TABLE 1:** Adsorption kinetic models and parameters for triclosan removal with NaAlg/MnS_0.05g_ bio-nanocomposite hydrogel.

Kinetic models	Parameters	Values	Adjusted *R* ^2^	*R* ^2^
	q_e_ (experimental) (mg/g)	85.7	-	-
Pseudo-first order	q_e_ (mg/g)	81.5	0.9702	**0.9735**
	k_1_ (min^-1^)	0.048		
Pseudo-second order	q_e_ (mg/g)	113.5	0.9944	**0.9944**
	k_2_ (mg/g.min)	0.0004		
Intra-particle diffusion	K_*i*_ (mg/g.min^1/2^)	10.4	0.8815	**0.8947**
	C	2.7		
Elovich	α (mg/g.min)	7.1	0.9294	**0.9373**
	$\beta_{e}$ (g/mg)	0.03		

Bold values indicate the R_2_ values of the determined kinetic models.

The experimental adsorption capacity was 85.7 mg/g and closely matched the calculated adsorption capacity for pseudo-first order model of 81.5 mg/g. These results show that pseudo-first order agreed with the experimental adsorption capacity, although it had a low *R* ^2^ value of 0.9735 compared to pseudo-second order with an *R* ^2^ value of 0.9944. The Elovich model is commonly used to analyse the adsorption kinetics because it describes a chemisorption mechanism occurring in the adsorption process (Kajjumba et al., 2018). Higher α values suggest faster initial adsorption rates; therefore, this means faster adsorption kinetics in the system (Al-Harby et al., 2021). In contrast, the β constant relates to surface charge coverage and activation energy for chemisorption. A higher β value indicates a lower activation energy barrier for adsorption (Al-Niemi, 2019). The α value for the study was 7.1 mg/g.min, which was greater than 1 mg/g.min signifying a high adsorption rate. While a β value <1 g/mg indicates a greater propensity for adsorption rather than desorption in the system. The β for the study was 0.03 g/mg, which is found to be less than 1 g/mg. This suggests that once the triclosan molecules are attached to NaAlg/MnS_0.05g_ bio-nanocomposite hydrogel, their likelihood of being released back into the solution is minimal.

##### 3.1.11.2 Adsorption isotherms

**FIGURE 10:**
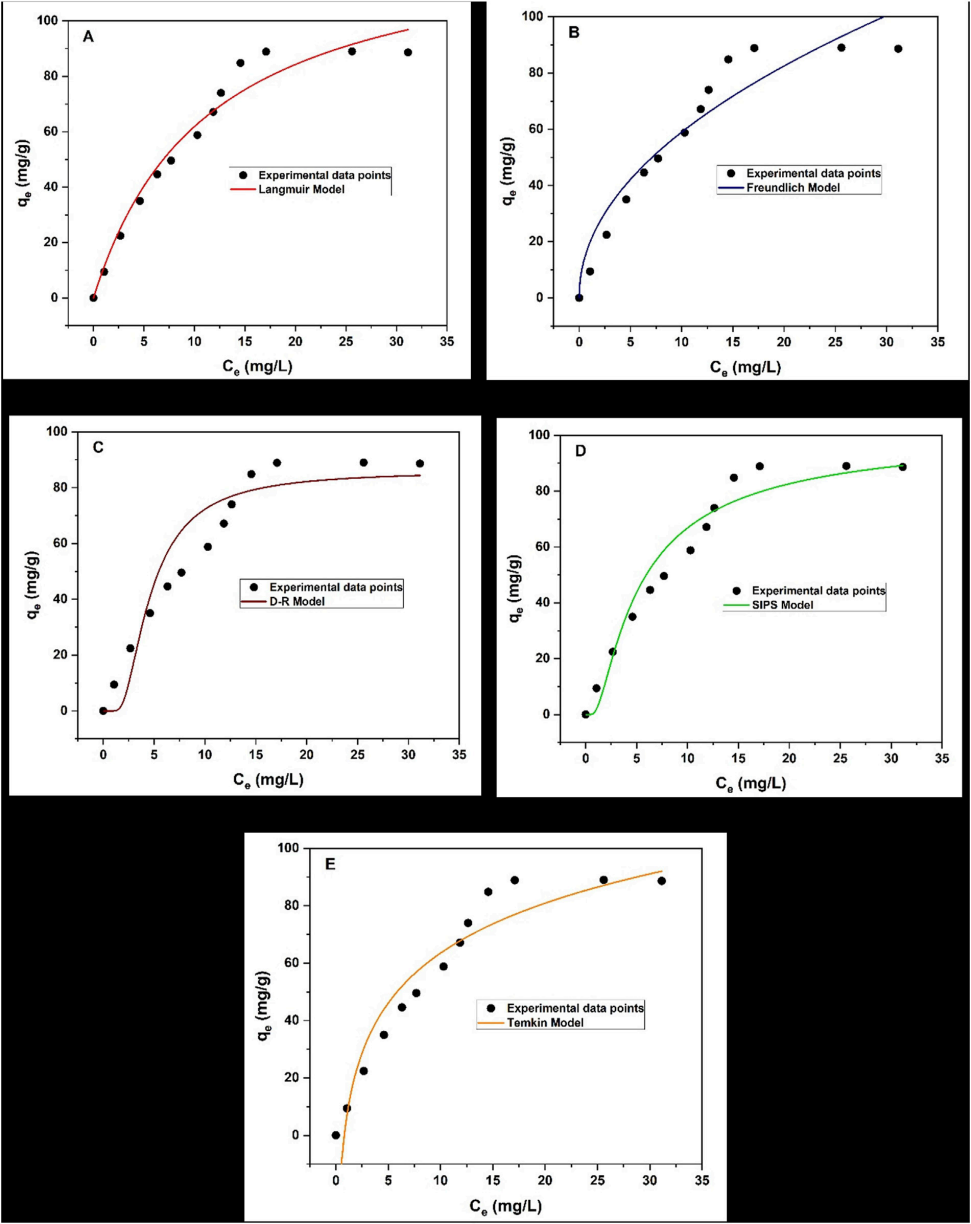
Adsorption Isotherms for NaAlg/MnS_0.05g_ bio-nanocomposite hydrogel on the removal of triclosan **(a)** Langmuir model, **(b)** Freundlich model, **(c)** D-R model, **(d)** Sips model, and **(e)** Temkin model. Experimental conditions: Sample volume, mass of adsorbent, pH, contact time and initial concentration were 35.1 mL, 11.5 mg, 8.68, 30 min and 60 mg/L.

This study examined isotherm models for the adsorption of triclosan onto the surface of NaAlg/MnS_0.05g_ bio-nanocomposite hydrogel. Figures 10a–e displays the adsorption isotherm results of this study and the fitted models used at each chart for Langmuir, Freundlich, Dubinin-Radushkevich (D-R), Sips, and Temkin models. When the initial concentration of triclosan was increased, a greater adsorption capacity was observed.

According to the data presented in Table 2, the removal of triclosan using NaAlg/MnS_0.05g_ bio-nanocomposite hydrogel followed the order of Freundlich > Sips > Langmuir > Temkin > D-R models. The Temkin model assumes that there is a linear decrease in the adsorption heat of all molecules when the surface of the adsorbent increases coverage. Furthermore, the model assumes a uniform distribution of binding energies up to a maximum binding energy (K_T_), implying that the adsorbent can consistently bind the adsorbate molecules. This model also considers the indirect interactions between adsorbate molecules on the adsorbent surface, which might impact the adsorption process (Shikuku and Jemutai-Kimosop, 2020; Piccin et al., 2011; Kalam et al., 2021).

**TABLE 2:** Adsorption isotherm model and parameters for triclosan on NaAlg/MnS_0.05g_ bio-nanocomposite hydrogel.

Adsorption models	Parameters	Values	Adjusted *R* ^2^	*R* ^2^
	q_e_ (experimental)	88.98	-	-
Langmuir	q_max_ (mg/g)	132.18	0.9665	**0.9693**
	K_L_ (L/g)	0.088		
Freundlich	K_F_ (L/mg)	19.31	0.9796	**0.9797**
	n	2.06		
Dubinin-Radushkevich (D-R)	q_max_ (mg/g)	86.01	0.7795	**0.7797**
	ε	19.19		
Sips	q_max_ (mg/L)	102.28	0.9793	**0.9793**
	K_s_ (L/g)	23.60		
	n_s_	100.79		
Temkin	K_T_ (L/mg)	1.25	0.9495	**0.9496**
	b (J/mol)	25.13		

Bold values indicate R_2_ values of the determined adsorption models.

The study favoured two isotherm models, which were the Freundlich and Sips models, as they had the highest *R* ^2^ values of 0.9797 and 0.9793, respectively. This was similar to a study done by (Kaur et al., 2019), where the removal of triclosan in water using activated carbon favoured two isotherm models (Freundlich and Langmuir). The Freundlich model characterises the adsorption process on surfaces with different affinities and energy distributions. This means that the adsorption process is multilayer and heterogeneous (Vigdorowitsch et al., 2021; Khayyun and Mseer, 2019; Kalam et al., 2021). K_F_ measures the adsorption capacity of the adsorbent, and higher K_F_ > 1 suggests that NaAlg/MnS_0.05g_ bio-nanocomposite hydrogel has a greater adsorption capacity for the triclosan analyte (Khayyun and Mseer, 2019). The Sips isotherm is a cohesion of Langmuir and Freundlich isotherms and explains both monolayer and multilayer adsorption patterns (Tzabar and Brake, 2016; Abin-Bazaine et al., 2022). When the concentration of NaAlg/MnS_0.05g_ bio-nanocomposite hydrogel is low, the Sips isotherm takes characteristics of the Freundlich isotherm. It describes the adsorption process as multilayer, while at a high NaAlg/MnS_0.05g_ bio-nanocomposite hydrogel concentration, it follows the behavioural patterns of Langmuir isotherm (Tzabar and Brake, 2016). When the values of n_s_ are greater than 1, the Sips model behaves homogenously and approaches the Langmuir isotherm characteristics. On the other hand, when n_s_ is less than 1, the Sips model moves closer to a heterogenous surface and approaches the Freundlich isotherm (Mehrvarz et al., 2017). The D-R isotherm model can calculate the adsorbent’s characteristic porosity and the apparent free energy of adsorption per mole of the adsorbate as it migrates to the surface from an unlimited distance in the solution (Yap and Priyaa, 2019; Batool et al., 2018). When the ε value of the D-R model is less than 8 kJ/mol, it suggests a physisorption mechanism occurs. When the ε value is greater than 16 kJ/mol, it indicates a chemisorption mechanism (Hu and Zhang, 2019). Therefore, the ε value in this study is greater than 16 kJ/mol and suggests that a chemisorption mechanism dominates the adsorption process.

##### 3.1.11.3 Adsorption thermodynamics

This study also investigated the thermodynamic properties and the effect of temperature on the adsorption of triclosan by NaAlg/MnS_0.05g_ bio-nanocomposite hydrogel. The adsorption process was carried out using optimum conditions and only varying the temperatures in a range of 298–318 K. The ∆H and ∆S values were estimated from Supplementary Figure S5, while ∆G was calculated from Equation 6. Table 3 presented the thermodynamic parameters for the adsorption of triclosan onto NaAlg/MnS_0.05g_ bio-nanocomposite hydrogel. The positive ∆H indicates that the adsorption process is endothermic. An endothermic adsorption process suggests that an increase in temperature correlates with an increase in the quantity of triclosan that can be adsorbed (Mustapha et al., 2019). Higher temperatures supply increased energy to surmount activation barriers, thereby enhancing the adsorption process. A positive ∆S shows increased randomisation at the solid/liquid interface. This implies that there is far greater freedom of movement or a greater number of alternative arrangements than the reactants (Fosso-Kankeu et al., 2017). Results revealed that ∆G (−17.804 to −21.850 kJ/mol) was negative throughout the study, indicating that the adsorption process was spontaneous and energetically favoured.

**TABLE 3:** Thermodynamic parameters of NaAlg/MnS_0.05g_ bio-nanocomposite hydrogel on the removal of triclosan.

		∆G (kJ/mol)				
∆H (kJ/mol)	∆S (J/Kmol)	298 K	303 K	308 K	313 K	318 K
44.043	207.018	−17.804	−18.472	−19.734	−20.753	−21.850

##### 3.1.11.4 Adsorption mechanism

This study employed the FTIR analysis to compare the functional groups for NaAlg/MnS_0.05g_ bio-nanocomposite hydrogel before and after the adsorption of triclosan to elucidate the adsorption mechanism involved. Supplementary Figures S6a, b showed several new peaks on NaAlg/MnS_0.05g_ bio-nanocomposite hydrogel after triclosan adsorption. A peak at 1,625 cm^-1^, which is due to the stretching vibration of carboxyl groups on NaAlg/MnS_0.05g_ bio-nanocomposite hydrogel (after), is broader than that of NaAlg/MnS_0.05g_ bio-nanocomposite hydrogel (before). It was noticeable in Supplementary Figure S6b that the carboxyl peak had a slight shift when compared to Supplementary Figure S6a, and this is due to the adsorption of triclosan onto NaAlg/MnS_0.05g_ bio-nanocomposite hydrogel. Following the adsorption of triclosan, there was a far more intense and broader peak at a stretching vibration of 1,408 cm^-1^, attributed to the carboxyl group on NaAlg/MnS_0.05g_ bio-nanocomposite hydrogel (after). Furthermore, two intense peaks appeared on NaAlg/MnS_0.05g_ bio-nanocomposite hydrogel (after) at 1,105 cm^-1^ and 1,032 cm^-1^, belonging to the C–O and O-H stretching vibration, respectively, which were far more intense and broader than NaAlg/MnS_0.05g_ bio-nanocomposite hydrogel (before). The C-O might be emanating from the carboxyl group and the COOH groups of the bio-composite hydrogels. This suggests that these functional groups are involved in binding with triclosan, and one possible mechanism is the O-H group forming hydrogen bonding with the OH group of triclosan. This FTIR study confirmed that NaAlg/MnS_0.05g_ bio-nanocomposite hydrogel interacts through the carboxyl, C-O, and O-H functional groups with triclosan through hydrogen bonding and π-π interaction as adsorptive mechanisms. Besides the use of functional groups, some adsorption mechanism processes may have occurred through electrostatic interactions, hydrophobic interactions, and physical entrapments occurring between NaAlg/MnS_0.05g_ bio-nanocomposite hydrogel and triclosan.

#### 3.1.12 Comparison with previous studies

A comparison between NaAlg/MnS_0.05g_ bio-nanocomposite hydrogel and other adsorbents for various parameters but mainly focused on the maximum adsorption capacity (mg/g) was done, as seen in Table 4. Most of the experiments were conducted at a pH range from five to 8.68, at which natural water systems are found. The results demonstrate that the maximum adsorption capacity (132.0 mg/g) of NaAlg/MnS_0.05g_ bio-nanocomposite hydrogel was higher than other adsorbents despite employing a small adsorbent in most studies. The adsorption isotherm and kinetic studies for this study favoured the Freundlich and Sips isotherms, while pseudo-second order favoured kinetic studies. The maximum adsorption capacity of this work was comparable to (Cusioli et al., 2021; Triwiswara, Lee, et al., 2020), 103.45 and 88.85 mg/g, respectively, as they had the closest results to the current work. Adsorption isotherm and kinetic studies for (Cusioli et al., 2021; Triwiswara, Lee, et al., 2020), favoured Langmuir and pseudo-first order for (Cusioli et al., 2021) and Langmuir and pseudo-second order for (Triwiswara, Lee, et al., 2020), respectively. The pH levels for (Cusioli et al., 2021) were pH 7 and for this study the pH levels were 8.68, which are comparable pH levels for freshwater systems found in the environment. Although the behavioural states of triclosan will be slightly different from each other at these pH levels, whereas at pH 7, triclosan primarily exists in its neutral phenolic state. This is due to its pKa, which varies from 7.9 to 8.14, being somewhat greater than the pH of 7. At pH 8.68, triclosan predominantly exists in its phenolate form, indicating a higher degree of ionisation and potential bioavailability. This is attributed to its pKa value, suggesting that at pH levels exceeding this value, the majority of triclosan molecules will carry a negative charge. Hydrogels possess a three-dimensional, highly porous structure and attractive functional groups (including carboxyl and O-H) that form hydrogen bonding with triclosan. This configuration significantly increases the available active sites for adsorption than conventional adsorbents such as activated carbon and others. The porous structure promotes the diffusion of triclosan into the hydrogel, thereby improving the overall adsorption efficiency (Zhang et al., 2021; Darban et al., 2022). These findings therefore demonstrated the high efficiency of NaAlg/MnS_0.05g_ bio-nanocomposite hydrogel in removing triclosan from aqueous solutions.

**TABLE 4:** Comparison of NaAlg/MnS_0.05g_ bio-nanocomposite with other adsorbents on the removal of triclosan.

Adsorbent	pH	Mass of adsorbent (mg)	Maximum adsorption capacity (mg/g)	Kinetic model	Isotherm model	References
MOM-Fe_3_O_4_	7	10.0	103.45	Pseudo-first order	Langmuir	Cusioli et al. (2021)
Carbon black	7	20.0	18.62	Pseudo-second order	Freundlich	Wang et al. (2022)
Thermally treated rice husks	5.6	200.0	72.70	Pseudo-first order	Langmuir	Triwiswara, Kang, et al. (2020)
Kenaf-derived biochar	6.3	77.4	13.03	Pseudo-second order	Langmuir	Cho et al. (2021)
Pam kernel shell	-	200.0	88.85	Pseudo-second order	Langmuir	Triwiswara, Lee, et al. (2020)
NaAlg/MnS_0.05g_ hydrogel	8.68	11.5	132.18	Pseudo-second order	Freundlich and Sips	This study

#### 3.1.13 Reusability and regeneration studies

**FIGURE 11:**
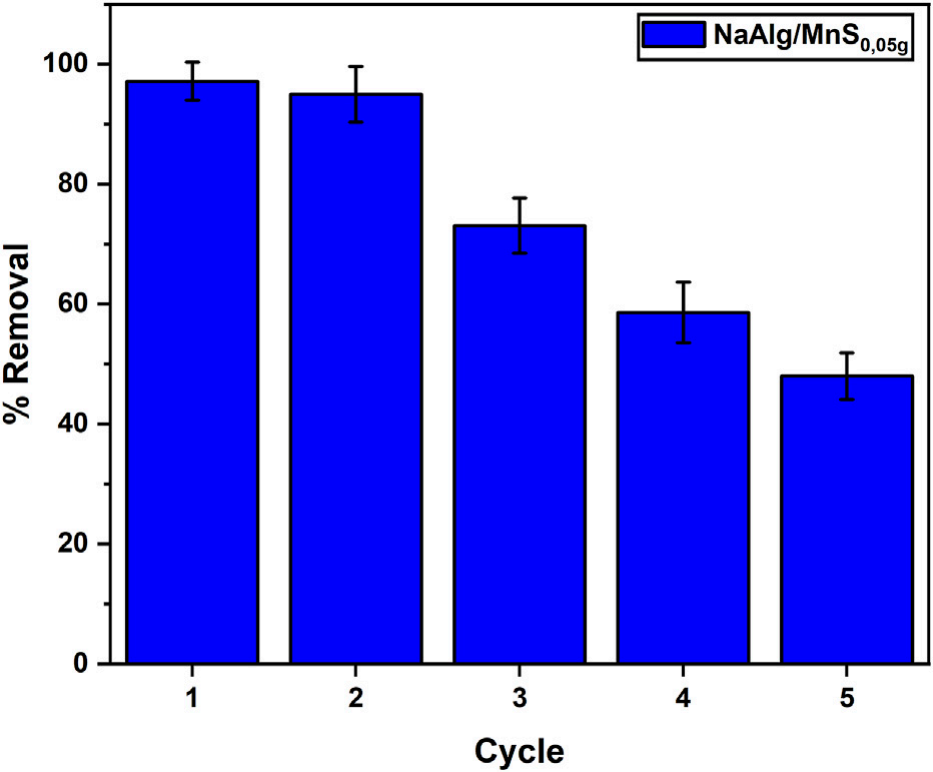
Reusability and regeneration studies for NaAlg/MnS_0.05g_ bio-nanocomposite hydrogel onto triclosan. Sample volume, mass of adsorbent, pH, contact time, initial concentration and desoption time were 35.1 mL, 11.5 mg, 8.68, 30 min, 1.0 mg/L and 2 h.

Repeatedly reusing an adsorbent reduces the overall costs related to the adsorption process. Adsorbents can be expensive; therefore, their reusability improves the economic feasibility of the treatment. This investigation utilised five cycles under optimal settings. The NaAlg/MnS_0.05g_ hydrogel was neutralised for reuse following each adsorption step and subsequently rinsed with 0.1M NaOH solution for 2 h, as per literature, and then rinsed with ultrapure water (Shoaib et al., 2023). The clearance rate of NaAlg/MnS_0.05g_ hydrogel exceeded 70% through the third cycle, but a steady reduction with an increase in the number of cycles was witnessed. This behavioural pattern was similar to a study by (Ko et al., 2024). The regular utilisation of nanocomposite hydrogels often leads to structural degradation. Mechanical stress during adsorption and desorption can cause micro-cracks and fractures in the hydrogel matrix. This degradation reduces the overall mechanical strength and stability of the material, rendering it less effective for subsequent usage cycles (Alcalde-Garcia et al., 2023). This may be attributed to the active sites and porous structure of the NaAlg/MnS_0.05g_ hydrogel, which were saturated with triclosan ions. The successful reuse of the adsorbent is considered significant, as seen in the data in Figure 11.

#### 3.1.14 Limitations of the study

NaAlg/MnS_0.05g_ bio-nanocomposite hydrogel beads exhibited the potential for the removal of triclosan in aqueous solutions owing to their adsorption properties. Nevertheless, there are limitations and scalability challenges linked to the study. NaAlg/MnS_0.05g_ bio-nanocomposite hydrogel beads can efficiently adsorb triclosan, however, their adsorption capabilities may be limited for extensive application or elevated concentrations. This may require large quantities of NaAlg/MnS_0.05g_ bio-nanocomposite hydrogels for efficient removal. The stability of NaAlg/MnS_0.05g_ bio-nanocomposite hydrogels under various environmental surroundings (such as low or extremely high pH levels) can be a challenge. Their durability under multiple cycles of use and regeneration is a factor to be considered, as seen in Figure 11. Despite the abundance and low cost of NaAlg/MnS_0.05g_ bio-nanocomposite hydrogel beads, scaling up the adsorbent production while preserving their effectiveness and cost-efficiency could be challenging. As production scales up, ensuring uniform material qualities becomes challenging. Discrepancies in batch-to-batch performance may arise despite the absence of observable alterations in the production process, impacting the efficacy of the adsorbent (Nazari et al., 2024). The simplicity of incorporating NaAlg/MnS_0.05g_ bio-nanocomposite hydrogels into current water treatment systems can vary depending on their mechanical characteristics and stability as per requirement.

#### 3.1.15 Environmental impact

The disposal of NaAlg/MnS_0.05g_ bio-nanocomposite hydrogel beads poses environmental challenges owing to their composition and probable ecological repercussions. These beads, were employed in water treatment management, integrate a biodegradable polymer such as sodium alginate with non-biodegradable manganese sulphide nanoparticles. Although biodegradable polymers are environmentally benign and decompose naturally, the included nanoparticles may last in the environment, resulting in possible toxicity and bioaccumulation. Inappropriate disposal of NaAlg/MnS_0.05g_ bio-nanocomposite hydrogel beads may release toxic compounds into aquatic environments, altering ecosystems and affecting microbial populations. To mitigate these impacts, safe disposal practices are essential such as secure landfilling, which is an alternative method, wherein NaAlg/MnS_0.05g_ bio-nanocomposite hydrogel beads can deposited in landfills engineered to inhibit leakage into groundwater. This approach is efficient for contaminants such as MnS NPs that cannot be biodegraded but necessitates meticulous monitoring to prevent environmental pollution (Malatji et al., 2021; Poorani et al., 2021).

## 4 Conclusion

In this work, pristine NaAlg hydrogel and NaAlg/MnS*x* bio-nanocomposite hydrogels prepared at distinct ratios of MnS NPs (0.02, 0.05, 0.1. 0.2 g) were successfully synthesised to remove triclosan effectively from water. The adsorbents were synthesised using sodium alginate, manganese sulphide nanoparticles, and calcium chloride (crosslinker) through the ionic gelation method. After that, the functional groups, morphology, elemental composition, thermal stability, surface charge, and crystallinity of these materials were successfully confirmed by SEM, TEM, EDX, FTIR, XRD, TGA, and zeta sizer. Swelling ratio (%) was also determined, and results showed an increase in mechanical strength in liquid up-take for NaAlg/MnS*x* bio-nanocomposite hydrogels compared to pristine NaAlg hydrogel. NaAlg/MnS_0.05g_ bio-nanocomposite hydrogel was selected as the best-performing adsorbent for the study since it had better removal recoveries. The adsorption of triclosan by NaAlg/MnS_0.05g_ best-fitted the Freundlich and Sips isotherm models as they both had higher and indistinguishable *R* ^2^ values. An experimental adsorption capacity of 88.98 (mg/g) was determined. The kinetic studies revealed that the adsorption process followed pseudo-second order and suggested that chemical reactions between the adsorbent and adsorbate in the adsorption process control the rate-limiting step. Thermodynamic studies revealed that ∆G was negative throughout the studied temperature range, indicating that the adsorption process was spontaneously and energetically favoured. A comparison with previous literature on NaAlg/MnS_0.05g_ bio-nanocomposite with other adsorbents on the removal of triclosan in water was done. Results demonstrated that NaAlg/MnS_0.05g_ bio-nanocomposite had a high maximum adsorption capacity compared to (Cusioli et al., 2021; Triwiswara, Lee, et al., 2020). The study points out the widespread problem of triclosan contamination in global water systems, revealing that its inadequate removal during wastewater treatment presents considerable risks to both aquatic ecosystems and human health, thereby emphasising the pressing necessity for stricter regulations and enhanced treatment processes to reduce its environmental impact.

## Data Availability

The original contributions presented in the study are included in the article/Supplementary Material, further inquiries can be directed to the corresponding author.
