# Supplementary material for: Effective adsorptive removal of triclosan from water using bio-nanocomposite hydrogel beads
Source: Front Chem. 2025 Apr 11;13:1547169. doi: 10.3389/fchem.2025.1547169 (PMC12022511; doi:10.3389/fchem.2025.1547169)
Supplement: Supplementary file 1 [file DataSheet1.docx]

Supplementary Material

# Supplementary Tables and Figures

**Tables**

Table S1: Gradient elution parameters on HPLC-DAD.

| Time [min] | A [%] | C [%] | Flow [mL/min] | Max Pressure limit [bar] |
| --- | --- | --- | --- | --- |
| 0.0 | 35.0 | 65.0 | 1.0 | 600 |
| 8.0 | 18.0 | 82.0 | 1.0 | 600 |
| 10.0 | 30.0 | 70.0 | 1.0 | 600 |

Table S2: Central composite design matrix

| **Standard Run** | **Parameters** | | | |
| --- | --- | --- | --- | --- |
|  | **MA (mg)** | **pH** | **SV (mL)** | **q_e_ (mg/g)** |
| **1** | 10.0 | 6.0 | 15.0 | 40.6 |
| **2** | 10.0 | 6.0 | 30.0 | 55.6 |
| **3** | 10.0 | 8.0 | 15.0 | 62.7 |
| **4** | 10.0 | 8.0 | 30.0 | 72.1 |
| **5** | 30.0 | 6.0 | 15.0 | 51.1 |
| **6** | 30.0 | 6.0 | 30.0 | 63.2 |
| **7** | 30.0 | 8.0 | 15.0 | 72.9 |
| **8** | 30.0 | 8.0 | 30.0 | 91.8 |
| **9** | 3.2 | 7.0 | 22.5 | 55.6 |
| **10** | 36.8 | 7.0 | 22.5 | 76.7 |
| **11** | 20.0 | 5.3 | 22.5 | 48.6 |
| **12** | 20.0 | 8.7 | 22.5 | 115.3 |
| **13** | 20.0 | 7.0 | 9.9 | 23.4 |
| **14** | 20.0 | 7.0 | 35.1 | 117.7 |
| **15 (C)** | 20.0 | 7.0 | 22.5 | 91.6 |
| **16 (C)** | 20.0 | 7.0 | 22.5 | 91.6 |
| **17 (C)** | 20.0 | 7.0 | 22.5 | 91.6 |
| **18 (C)** | 20.0 | 7.0 | 22.5 | 92.2 |

Table S3: Analysis of variance (ANOVA)

| **Factor** |  | | | | |
| --- | --- | --- | --- | --- | --- |
|  | **SS** | **df** | **MS** | **F** | **p** |
| **(1) MA (mg)(L)** | 512.24 | 1 | 512.239 | 1.370473 | 0.266467 |
| **(2) pH (L)** | 2960.82 | 1 | 2960.823 | 7.921554 | 0.016832 |
| **(3) SV (mL)(L)** | 3354.73 | 1 | 3354.734 | 8.975447 | 0.012169 |
| **1L by 2L** | 17.40 | 1 | 17.397 | 0.046546 | 0.833135 |
| **1L by 3L** | 5.41 | 1 | 5.408 | 0.014470 | 0.906422 |
| **2L by 3L** | 0.21 | 1 | 0.212 | 0.000566 | 0.981444 |

Table S4: Isotherm models fitted for the adsorption of triclosan onto NaAlg/MnS_0.05g_ bio-nanocomposite hydrogel.

| **Isotherm Models** | **Expression** | **Definitions** | **Model Assumptions** |
| --- | --- | --- | --- |
| **Langmuir** | $q_{e}=\frac{q_{max}K_{L}C_{e}}{1+ K_{L}C_{e}}$ | q_max_ is the maximum adsorption capacity (mg/g), and K_L_ is the Langmuir constant (L/mg). | The model presupposes that adsorption occurs exclusively up to a single layer of molecules on the surface of the adsorbent. Saturation coverage is achieved when all available sites are occupied, preventing any additional adsorption. |
| **Freundlich** | $q_{e}= K_{f}C_{e}^{\frac{1}{n}}$ | K*_f_* (L/mg) is the Freundlich constant, and 1/*n* is the heterogeneity factor. | The model assumes that the adsorbent surface is heterogeneous, which implies that it contains sites with distinct adsorption affinities and energies. As a result, the rates of adsorption at these sites vary. |
| **Temkin** | $q_{e}=\frac{RT}{b}ln(K_{T}C_{e})$ | b is related to the heat of adsorption (J/mol), R is the gas constant (8.314 J/mol.K), T is the absolute temperature (K), and K*_T_* is the Temkin equilibrium constant (L/g). | The model assumes that the adsorbent surface is uniformly distributed with heterogeneous binding sites, which enables the adsorption energy to vary across these binding sites. |
| **Dubinin-Radushkevich (D-R)** | $q_{e}=q_{{max}^{\exp-\beta\varepsilon^{2}}}$ | β is the D-R constant, ε = RTln(1+ $\frac{1}{C_{e}}$) is Polanyi's potential (kJ/mol). | It asserts that the adsorption energy allocation across the adsorbent's active sites adheres to a Gaussian distribution. This illustrates that distinct locations possess varying energies, influencing the interactions of gas molecules with them. |
| **Sips** | $q_{e}=\frac{q_{max}K_{s}C_{e}^{n_{s}}}{1+ K_{s}C_{e}^{n_{s}}}$ | K*_s_* is the Sips constant (L/mg), and n*_s_* is the Sips exponent describing either the homogeneity or heterogeneity of the adsorption process. | The model assumes that adsorption transpires on a heterogeneous surface, characterised by adsorption sites with diverse energy levels. It delineates a transition from Freundlich-like multilayer adsorption at low adsorbate concentrations to Langmuir-like monolayer adsorption at elevated concentrations, integrating elements of both models to depict a more accurate adsorption phenomenon across an extensive spectrum. |

Table S5: Adsorption kinetic models fitted for the removal of triclosan onto NaAlg/MnS_0.05g_ bio-nanocomposite hydrogel.

| **Kinetic models** | **Expression** | **Definitions** | **Model Assumptions** |
| --- | --- | --- | --- |
| **Pseudo-first order** | $q_{t}= q_{e}(1-e^{-k_{1}t})$ | k_1_ is the adsorption rate (min^-1^), and t (min) is the adsorption time. | The model posits that the adsorption rate is governed by the diffusion of the adsorbate across the adsorbent surface. |
| **Pseudo-second order** | $q_{t}= \frac{k_{2}q_{e}^{2}t}{1+k_{2}q_{e}t}$ | k_2_ is the adsorption rate (mg/g.min). | The model proposes that the adsorption rate is proportional to the square of the quantity of adsorbate already present on the adsorbent. This indicates a second order kinetic process, wherein interactions among adsorbed molecules might affect subsequent adsorption. |
| **Elovich** | $q_{t}= \frac{1}{\beta_{e}}\ln(1+ \alpha\beta_{e}t)$ | α (mg/g.min) is the initial adsorption rate at time t (min), and $\beta_{e}$ (g/mg) is a desorption constant related to the adsorbate layer on the adsorbent surface as well as the activation energy of the chemisorption process. | The Elovich model suggests that the adsorbent surface is energetically heterogeneous, indicating that distinct adsorption sites possess differing energy levels. This heterogeneity influences the interaction of adsorbates with the surface, resulting in varying activation energies for adsorption. |
| **Intra-particle diffusion** | $q_{t}= K_{i}t^{\frac{1}{2}}+C$ | K*_i_* is the rate constant (g/mg.min^0.5^), and C (mg/g) is a constant related to the boundary layer thickness. | The model assumes that the adsorption rate is predominantly governed by the diffusion of the adsorbate molecules via the adsorbate's pores. This indicates that when the adsorbate arrives at the surface, its ingress into the porous structure constitutes the rate-limiting stage in the entire adsorption. |

**Figures**

Figure S1: Zeta Potential (mV) of (a) pristine NaAlg hydrogel, (b) NaAlg/MnS_0.02g_ hydrogel, (c) NaAlg/MnS_0.05g_ hydrogel, (d) NaAlg/MnS_0.1g_ hydrogel, and (e) NaAlg/MnS_0.2g_ hydrogel.


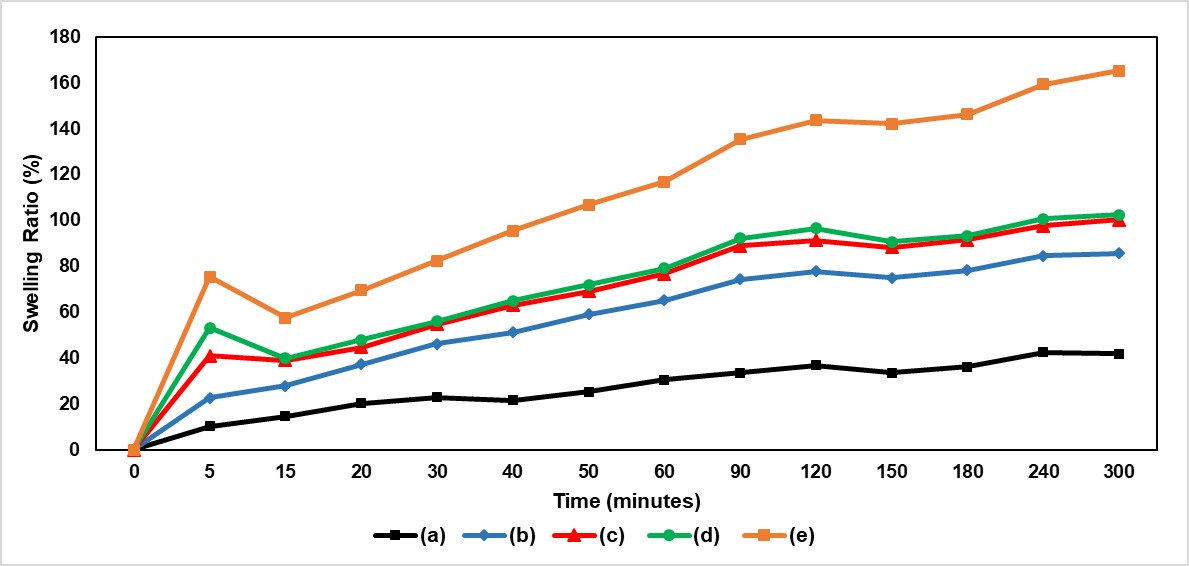


Figure S2: Swelling ratio (%) of (a) pristine NaAlg hydrogel, (b) NaAlg/MnS_0.02g_ hydrogel, (c) NaAlg/MnS_0.05g_ hydrogel, (d) NaAlg/MnS_0.1g_ hydrogel, and (e) NaAlg/MnS_0.2g_ hydrogel.


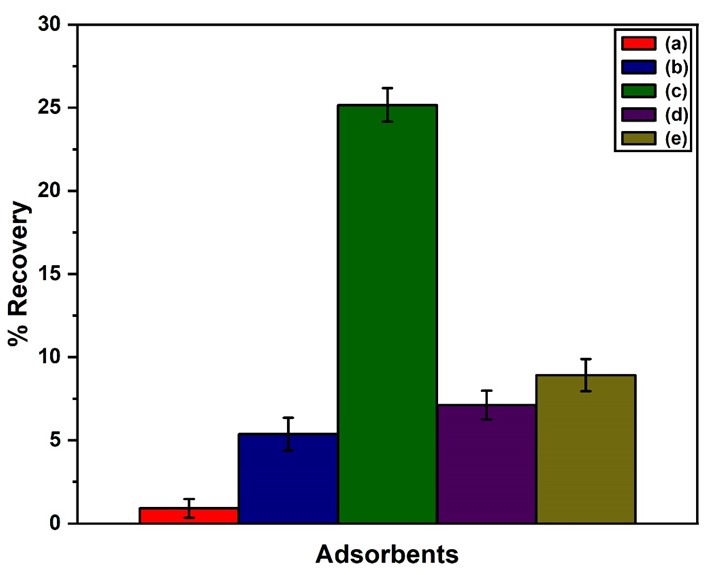


Figure S3: Percentage recovery (%) of (a) pristine NaAlg hydrogel, (b) NaAlg/MnS_0.02g_ hydrogel, (c) NaAlg/MnS_0.05g_ hydrogel, (d) NaAlg/MnS_0.1g_ hydrogel, and (e) NaAlg/MnS_0.2g_ hydrogel.


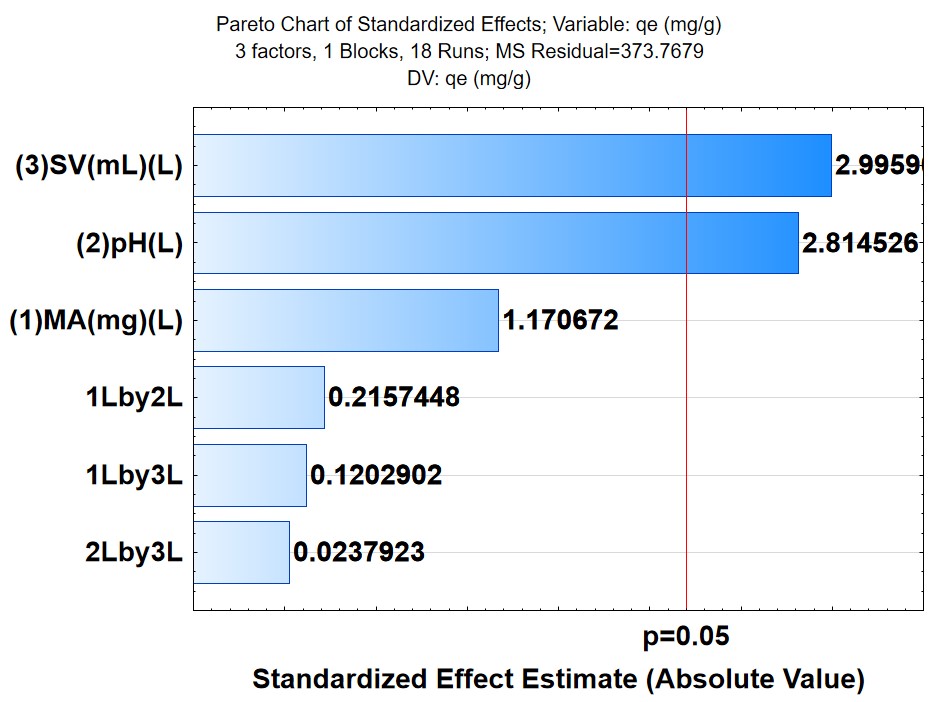


Figure S4: Pareto chart of standardised effects for the adsorption of triclosan.


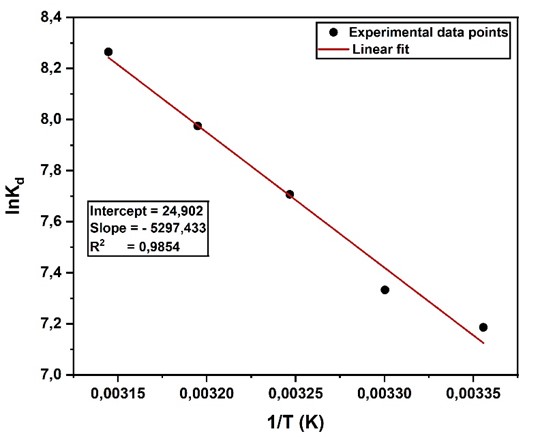


Figure S5: Van't Hoff plot to calculate thermodynamic parameters.


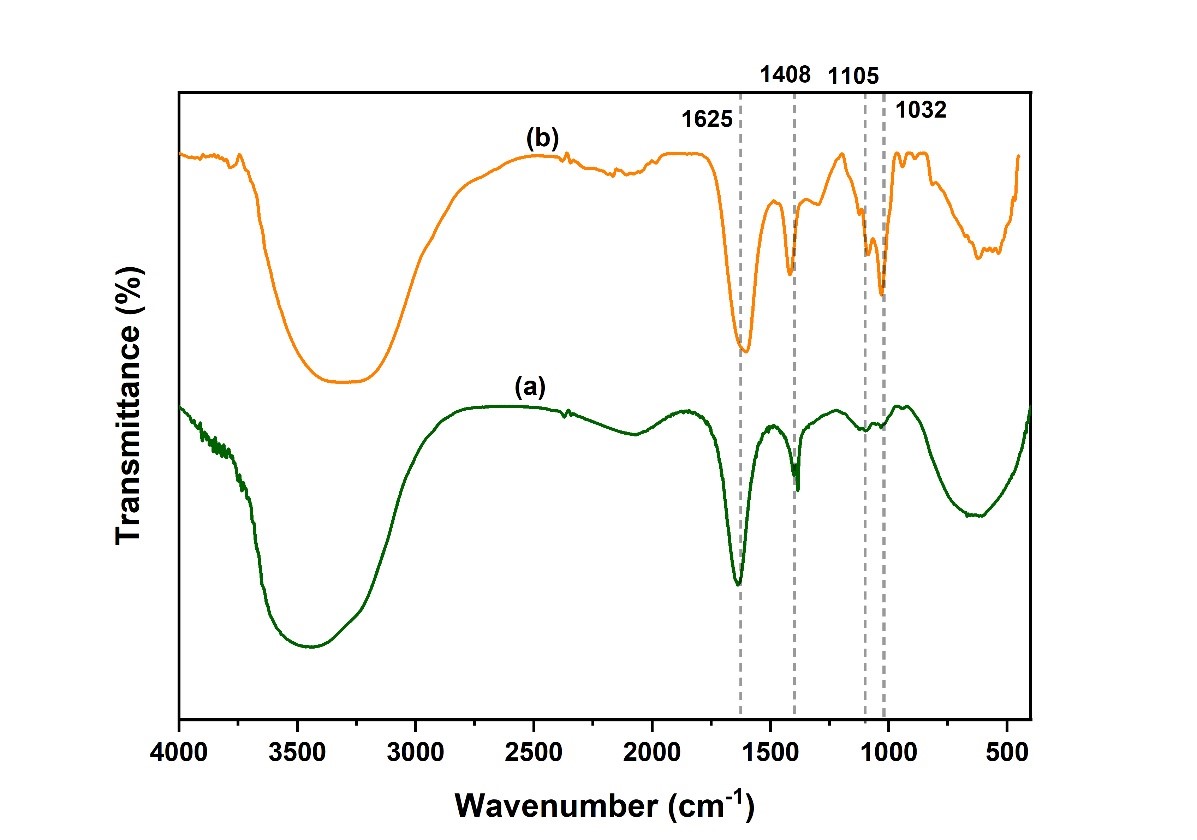


Figure S6: FTIR adsorption mechanism for (a) NaAlg/MnS_0.05g_ bio-nanocomposite hydrogel (before) and (b) NaAlg/MnS_0.05g_ bio-nanocomposite hydrogel (after).
